# Comparative effect of different surgical treatments for ovarian endometrioma on anti-Müllerian hormone levels: a systematic review and network meta-analysis

**DOI:** 10.1093/hropen/hoag019

**Published:** 2026-03-01

**Authors:** Konstantinos Nirgianakis, Dimitrios Rafail Kalaitzopoulos, Nikolaus Fadinger, Michael D Mueller, Chiara Gastaldon

**Affiliations:** Department of Obstetrics and Gynecology, University Hospital and University of Bern, Bern, Switzerland; Center for Gynecologic Surgery, Heraklion, Greece; Department of Obstetrics and Gynecology, Cantonal Hospital Schaffhausen, Schaffhausen, Switzerland; Department of Obstetrics and Gynecology, University Hospital and University of Bern, Bern, Switzerland; Department of Obstetrics and Gynecology, University Hospital and University of Bern, Bern, Switzerland; Institute of Social and Preventive Medicine (ISPM), University of Bern, Bern, Switzerland; Department of Neuroscience, Biomedicine and Movement Sciences, Section of Psychiatry, WHO Collaborating Centre for Research and Training in Mental Health and Service Evaluation, University of Verona, Verona, Italy

**Keywords:** endometriosis, ovarian endometrioma, ovarian reserves, AMH, AFC

## Abstract

**STUDY QUESTION:**

Which surgical technique for endometrioma has the least detrimental effect on ovarian reserves?

**SUMMARY ANSWER:**

Drainage with hemostatic sealants and cystectomy with ovarian suturing were associated with relatively smaller anti-Müllerian hormone (AMH) declines 3–6 months postoperatively; however, further research assessing fertility and long-term outcomes is needed to clarify the safety and efficacy of these techniques.

**WHAT IS KNOWN ALREADY:**

Ovarian endometriomas are common in women of reproductive age and can affect their ability to have a pregnancy. Surgical removal of the endometrioma is one of the strategies to favor pregnancy, with different possible surgical techniques. However, damage to the ovary can result in lower ovarian reserve. To date, it is uncertain what surgical technique provides the best balance between efficacy and safety.

**STUDY DESIGN, SIZE, DURATION:**

We conducted a systematic review and network meta-analysis (NMA), searching PubMed, Embase, and Cochrane Register of Trials for randomized controlled trials (RCTs) that compared the impact of different surgical techniques for endometrioma on ovarian reserves (measured with the AMH level or antral follicle count (AFC)) from inception to June 2024. All relevant peer-reviewed studies were included.

**PARTICIPANTS/MATERIALS, SETTING, METHODS:**

The primary outcome was ovarian reserve, measured by the change in AMH levels (ng/ml) at 3–6 months after the treatment. Secondary outcome was the change in AFC at 3–6 months after the surgery. We assessed the quality of included studies with the Cochrane risk of bias tool 2 and performed an NMA, comparing the head-to-head effect of different surgical strategies, calculating mean differences (MDs) and 95% CIs.

**MAIN RESULTS AND THE ROLE OF CHANCE:**

21 RCTs with 1519 participants, comparing 8 different surgical techniques (cystectomy with ovarian suturing, cystectomy with hemostatic sealants, cystectomy with tranexamic acid, cystectomy alone, drainage with hemostatic sealants, drainage alone, laser ablation, and transvaginal sclerotherapy) were included in the systematic review and 17 studies in the NMA. Regarding AMH at 3–6 months after surgery, drainage with hemostatic sealants (MD: 0.96; 95% CI: 0.60–1.33; high level of certainty), cystectomy with ovarian suturing (MD: 0.69; 95% CI: 0.39–0.98; moderate certainty), and cystectomy with hemostatic sealants (MD: 0.37; 95% CI: 0.12–0.61; low certainty) resulted in higher values of AMH compared with cystectomy alone. Regarding AFC at 3–6 months after surgery, laser ablation showed higher values of AFC (MD: 2.30; 95% CI: 0.20–4.40) compared with cystectomy alone at 3–6 months after surgery, followed by cystectomy with ovarian suturing (MD: 1.88; 95% CI: 0.98–2.79). The overall risk of bias of the included studies was low. The Confidence in Network Meta-Analysis (CINeMA) assessment showed that the only comparison with high certainty of evidence is the comparison between cystectomy and drainage with hemostatic sealants, followed by moderate certainty for the comparison between cystectomy and cystectomy with ovarian suturing and between cystectomy and laser, while the remaining comparisons had a low certainty of evidence.

**LIMITATIONS, REASONS FOR CAUTION:**

The heterogeneity among the included studies, due to differences in the timing of AMH measurement, the localization and size of endometriomas, and the varying surgical techniques and expertise, is the main limitation of this study. In addition, further reasons for caution are the short follow-up period, the lack of data regarding pregnancy rates, and specific interventions such as sclerotherapy.

**WIDER IMPLICATIONS OF THE FINDINGS:**

Considering the estimated effect sizes and certainty of evidence for both AMH and AFC, cystectomy with ovarian suturing and drainage with hemostatic sealants were associated with the least negative impact on ovarian reserve markers. Drainage with hemostatic sealants showed associations with higher short-term AMH preservation; however, this is based on limited data, primarily from a single RCT, and more evidence from different centers is needed to assess effectiveness, recurrence risk, and potential adverse effects such as inflammation or fibrosis from allogenic materials. Given the large number of patients operated on for endometriomas globally, we urgently call for well-powered, multicentric RCTs to compare reproductive outcomes across surgical techniques, including those associated with relatively higher AMH in our analysis.

**STUDY FUNDING/COMPETING INTEREST(S):**

This research did not receive any specific grant from funding agencies in the public, commercial, or not-for-profit sectors. The authors report no relevant conflict of interest.

**TRIAL REGISTRATION NUMBER:**

CRD42021238909.

WHAT DOES THIS MEAN FOR PATIENTS? Ovarian endometrioma, or ‘chocolate cyst’, is a type of ovarian cyst that is common in women of reproductive age and can affect their ability to become pregnant. Surgical removal of an endometrioma is often reasonable; however, the procedure can damage ovarian tissue and reduce the ovarian reserve. This study compared different surgical techniques for ovarian endometrioma and evaluated their impact on ovarian reserve. The techniques associated with the least reduction in ovarian reserve were cyst removal, followed by suturing of the remaining ovarian tissue and cyst drainage combined with hemostatic sealants. Given the high number of patients undergoing surgery for endometriomas worldwide, there is an urgent need for well-powered, multicenter randomized controlled trials to compare reproductive outcomes between surgical techniques.

## Introduction

Endometriosis is a highly prevalent estrogen-dependent gynecological disorder of reproductive-aged women, characterized by the growth of endometrial-like lesions outside the uterine cavity (Bulun, 2009; Gylfason *et al.*, 2010). The disease phenotype varies depending on the area/organ in which endometriosis lesions grow. When the ovary is affected, it usually creates an ovarian cyst lined with endometrial-like tissue and containing fluid from the accumulation of blood debris. This is called ovarian endometrioma, and it affects up to 55% of patients with endometriosis (Liu *et al.*, 2007). Symptoms include endometriosis-associated pelvic pain, pelvic fullness, and infertility, often leading to a significant reduction in quality of life (Zondervan *et al.*, 2020).

Different therapeutic options for endometrioma are available, chosen according to the specific patient’s characteristics such as age, symptoms, fertility, endometrioma size, and ultrasound-image. According to the most recent guidelines, when surgical therapy is indicated, laparoscopic cystectomy should be preferred to laparoscopic drainage and coagulation (Kalaitzopoulos *et al.*, 2021; Becker *et al.*, 2022), as it may decrease the risk of recurrence and pelvic pain in addition to an increased chance of spontaneous pregnancy (Porpora *et al.*, 2010; Carmona *et al.*, 2011). However, the main controversy associated with cystectomy is that it may damage or remove healthy ovarian cortex and follicles, leading to an adjunctive decrease of the impaired ovarian reserve following the procedure (Raffi *et al.*, 2012; Muzii *et al.*, 2018). Therefore, other surgical options, such as ablation by laser or plasma and sclerotherapy, have been developed with the aim to mitigate the ovarian reserve damage.

Evidence regarding the optimal intervention to minimize ovarian damage remains limited and inconsistent. A recent meta-analysis (Cohen *et al.*, 2017) suggested that sclerotherapy is a promising alternative to cystectomy for managing ovarian endometrioma. However, prior studies and meta-analyses have typically compared only two interventions at a time—most commonly cystectomy versus another treatment—without offering direct head-to-head comparisons among all available options. Given the variety of interventions for endometrioma and the ongoing uncertainty about which approach best preserves ovarian reserve, it is crucial to assess their comparative effectiveness comprehensively. Therefore, we conducted a network meta-analysis (NMA) that, for the first time, provides direct head-to-head comparisons of all surgical techniques using anti-Müllerian hormone (AMH) and antral follicle count (AFC) as markers of ovarian reserve. Additional outcomes evaluated included pregnancy and recurrence rates.

## Methods

We followed the Preferred Reporting Items for Systematic Reviews and Meta-Analyses (PRISMA) extension statement for network meta-analyses. The study protocol was registered in PROSPERO (registration number: CRD42021238909).

### Inclusion criteria

We performed a systematic review and NMA, including randomized controlled trials (RCTs) that compared the impact on ovarian reserves (using AMH or AFC) of different surgical treatments for unilateral or bilateral endometriomas. Studies with any type of surgical treatment, defined as an invasive procedure aiming to treat the endometrioma, i.e. cystectomy, modified cystectomy, ablation by laser or plasma, sclerotherapy, and draining, were eligible. We included participants with ovarian endometriomas of any age or ethnicity, as long as they were pre-menopausal. Studies with more than one treatment for the same patient were excluded. We excluded other types of studies (cohort, case–control, quasi-randomized) and reviews. Non-randomized studies were excluded from this NMA as meta-analyses of mixed study design is not suggested. When addressing efficacy and tolerability of interventions, RCTs and meta-analyses of them represent the most reliable and rigorous type of evidence (Murad *et al.*, 2016), as they are the only type of study that can infer causality, meaning that the differences in the outcome are related to a causal effect of the intervention. For the scope of this NMA, we limited the inclusion criteria to reduce heterogeneity between studies, avoid confounders and selection bias, and infer causality.

### Search

We systematically searched PubMed, Embase, and the Cochrane Register of Trials from inception until 1 June 2024. Combinations of the terms endometrioma, cystectomy, laser ablation, drainage, plasma, sclerotherapy, cystotomy, surgical treatment, and expectative were used (Supplementary File S1). We additionally searched the reference sections of relevant publications, key journals, and abstracts from major meetings in the field. Two investigators (D.R.K. and N.F.) screened the titles and abstracts of all records retrieved, then the full texts of eligible studies for inclusion, and any discrepancy was resolved by consultation with a third investigator (K.N.).

### Outcomes, data extraction, and evaluation of study risk of bias

Two reviewers (D.R.K. and N.F.) independently extracted relevant data from each included study using a pre-defined Excel extraction form. Data extracted included general characteristics of the study (author, year of publication, country, intervention, number of patients included, AMH kit used, and duration of follow-up), baseline characteristics (age, BMI, and size of endometrioma), and outcomes (mean endpoint and/or mean change levels of AMH and AFC in three or less and six or more months, recurrence of endometrioma, and number of pregnancies after surgical treatment). We contacted the corresponding authors of the studies via e-mail for missing information. Specifically, we contacted four authors (Giampaolino *et al.*, 2015; Shaltout *et al.*, 2019; Park *et al.*, 2021; Ghasemi Tehrani *et al.*, 2022) for missing data, and we received only the data from one study group (Shaltout *et al.*, 2019).

The primary outcome was ovarian reserve, measured by the change in AMH levels (ng/ml) at 3–6 months after the treatment. The secondary outcome was the change in AFC at 3–6 months after the surgery. We also extracted information on the rate of endometrioma recurrence, calculated as the number of patients who experienced a return of the endometrioma after surgery divided by the total number of patients and the rate of pregnancy at any time point after the surgery.

Two reviewers (D.R.K. and N.F.) independently assessed the risk of bias using the RoB2 Tool, classifying studies into three categories: ‘low risk of bias’, ‘high risk of bias’, and ‘some concerns’ (Sterne *et al.*, 2019). Any discrepancy was resolved through a discussion with a third investigator (K.N.).

### Data synthesis and evaluation of the confidence in the evidence

We performed a standard pairwise random-effects meta-analysis for each comparison and for each outcome, an NMA in a frequentist framework. For continuous outcomes, we pooled mean differences (MDs) and SD with 95% CIs between treatment arms after surgical treatment, as all studies were expected to employ the same unit of measure for hormonal blood levels. For studies reporting median and interquartile range, we estimated means and SDs before pooling MDs (Wan *et al.*, 2014; Luo *et al.*, 2018).

For the primary and secondary continuous outcomes (AMH and AFC), we used data provided at either 3 or 6 months. For studies that provided both time points, we calculated the mean between the two values (Younis *et al.*, 2022). As a subgroup analysis, we separately analyzed studies reporting AMH levels 3 and 6 months after surgery, in order to verify the consistency between these two time points. An NMA was not possible for longer-term follow-up as the majority of the included studies did not report these outcomes.

For pairwise meta-analyses, we assessed heterogeneity by visual inspection of forest plots and by *I*^2^ statistics. For the NMA of each outcome, common heterogeneity across all comparisons was assumed and estimated in each network. For each outcome, we assumed a common heterogeneity variance (τ^2^) across comparisons. The magnitude of heterogeneity was judged by comparing τ^2^ to its empirical distribution and by considering the width of the prediction intervals. Statistical inconsistency was evaluated using the SIDE test for each comparison and the design-by-treatment interaction test for the overall network. For each outcome, we produced a treatment hierarchy by employing the *P*-score, which represents a probabilistic approach similar to the means of surface under the cumulative ranking curve (SUCRA) (Mbuagbaw *et al.*, 2017).

To assess the plausibility of the transitivity assumption, we extracted potential effect modifiers (i.e. BMI, age, baseline AMH levels, and size of endometrioma) and compared their distribution across comparisons in the network. To explore potential sources of heterogeneity and inconsistency, we also planned network meta-regressions for baseline BMI, mean age, and size of the endometrioma. Moreover, we conducted a *post-hoc* subgroup analysis based on endometrioma size, using a clinically recognized cut-off (<5 cm vs >5 cm), given that surgical management of larger endometriomas may have a different impact on ovarian reserve compared to smaller lesions. Additionally, we performed a separate subgroup analysis according to endometrioma laterality, distinguishing between studies that included only women with unilateral endometriomas and those with a mixed population of unilateral and bilateral cases. This distinction was made because surgical treatment of bilateral endometriomas may have a more pronounced detrimental effect on ovarian reserves.

To verify the robustness of the results, we performed sensitivity analyses by excluding studies with an overall assessment of high risk of bias and studies for which we imputed the data.

Frequentist network and pairwise meta-analyses were performed with the netmeta, version 2.8-2 and meta, version 6.5-0 packages (Freiburg, Germany). The confidence in the NMA estimates was evaluated for the primary outcome with the Confidence in Network Meta-Analysis (CINeMA) framework, evaluating the certainty of evidence through these items: ‘within study bias’, ‘reporting bias’, ‘indirectness’, ‘imprecision’, ‘heterogeneity’, and ‘incoherence’.

## Results

### Description of included studies

**Figure 1.**
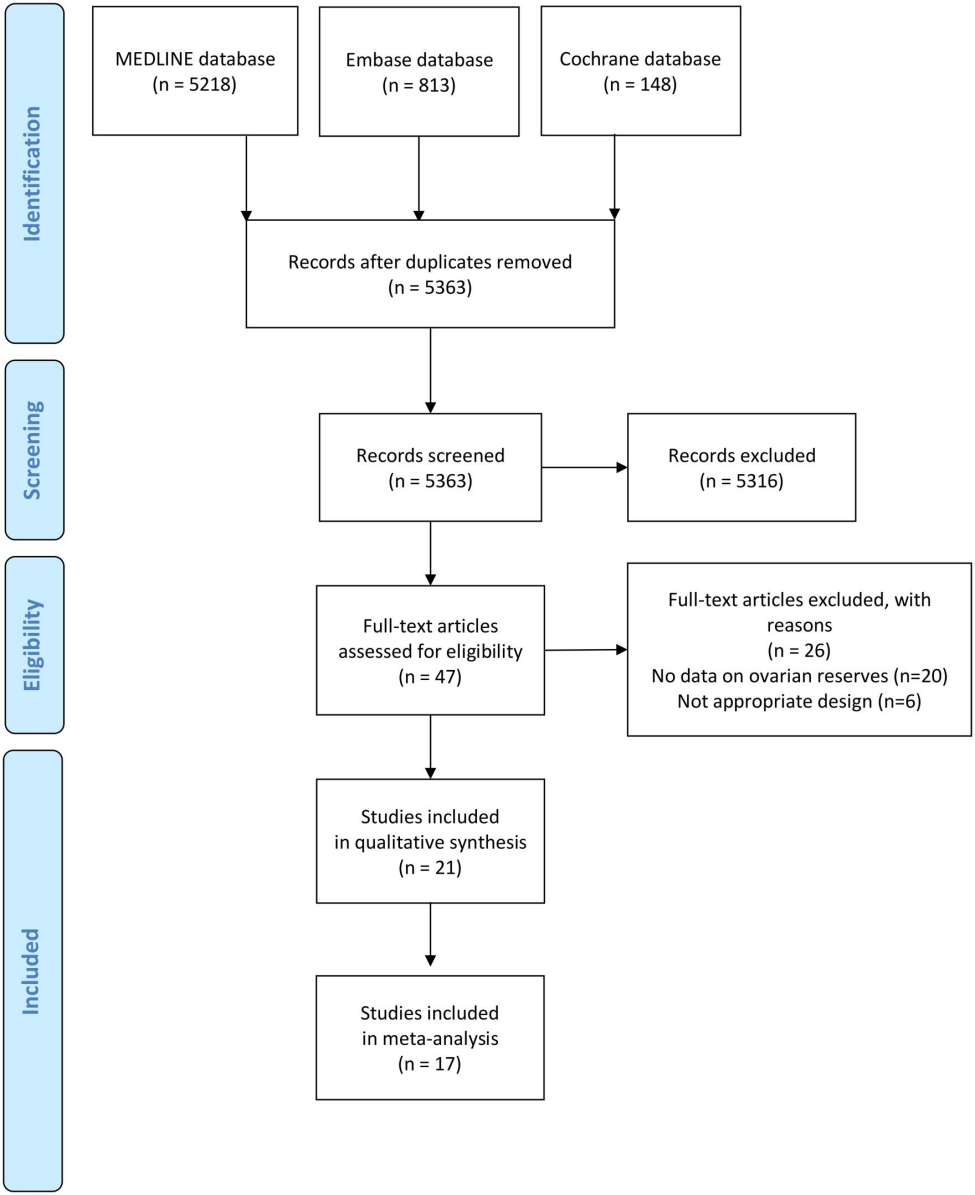
Flowchart of study selection.

We identified 6179 references published from 2012 to 1 June 2024 through the literature search. During the screening process, studies were excluded after the initial search if they were not relevant or were not RCTs. After screening of titles and abstracts, we assessed 47 full-texts and included 21 studies with 1519 participants in the systematic review and 17 studies in the NMA (Tsolakidis *et al.*, 2010; Coric *et al.*, 2011; Ferrero *et al.*, 2012; Sönmezer *et al.*, 2013; Ghafarnejad *et al.*, 2014; Tanprasertkul *et al.*, 2014; Giampaolino *et al.*, 2015; Asgari *et al.*, 2016; Zhang *et al.*, 2016; Candiani *et al.*, 2018; Choi *et al.*, 2018; Chung *et al.*, 2019; Rouholamin and Ahmadpour-Ghazvini, 2019; Shaltout *et al.*, 2019; Sweed *et al.*, 2019; Akkaranurakkul *et al.*, 2021; Javaheri *et al.*, 2021; Park *et al.*, 2021; Alborzi *et al.*, 2022; Araujo *et al.*, 2022; Ghasemi Tehrani *et al*., 2022). Several studies were excluded because they reported ovarian reserve solely based on ovarian volume, some applied the same surgical technique to both comparison groups, and one study employed two different surgical techniques within the same patients. Details are provided in the PRISMA flowchart (Fig. 1), in the list of excluded studies with reason (Supplementary Table S1), and in the tables with the characteristics of the included studies (Table 1; Supplementary Table S2). The mean age of participants was 30.08 (SD = 2.12) years, the mean BMI was 23.05 (SD = 1.89) kg/m^2^, and the mean endometrioma size was 5.79 (SD = 1.89) cm. The studies included eight different surgical techniques: cystectomy with ovarian suturing (ST), cystectomy with hemostatic sealants (HS), cystectomy with tranexamic acid (TXA), cystectomy alone, drainage with HS, drainage alone, laser ablation, and transvaginal sclerotherapy. These techniques that have been previously described and suggested for endometriomas are summarized in Table 2. Studies that used HS after cystectomy were categorized as cystectomy + HS, while studies that used ST after cystectomy were categorized as cystectomy + ST. Similarly, studies that used HS after drainage of the ovary were categorized as drainage + HS. The only study that compared transvaginal sclerotherapy to laparoscopic cystectomy reported discrepant results in the abstract and the manuscript, and it was unclear if the follow-up time was 6 weeks or 6 months after surgery. An effort to resolve the issue was made by contacting the corresponding author via email, but we received no response, so the study was not included in the NMA (Ghasemi Tehrani *et al.*, 2022). The detailed description of each intervention and its classification in this network is presented in Supplementary Table S3. Although the number of studies for each comparison was low, we did not find any clear violation of the transitivity assumption (Supplementary Fig. S1).

**Table 1.** Characteristics of randomized controlled trials included in network of AMH.

Characteristics	AMH network
Number of studies	17
Number of patients included	1314
	**Mean (SD)**
Age (years)	30.08 (2.12)
BMI (kg/m^2^)	23.05 (1.89)
Size (cm)	5.79 (1.89)
Baseline AMH baseline (ng/ml)	3.58 (1.15)
Localization of endometrioma	
Only unilateral	10 (58.8)
Only bilateral	1 (5.8)
Unilateral or bilateral	6 (35.3)

**Study follow-up duration**	**N of studies (%)**
3 months	13 (76.4)
6 months	8 (47)

**Type of comparison**	**N of studies (%)**
Cystectomy alone vs cystectomy + HS	9 (52.9)
Cystectomy alone vs Cystectomy + ST	6 (35.2)
Cystectomy alone vs laser	2 (11.8)
Cystectomy alone vs cystectomy + TXA i.v.	1 (5.8)
Cystectomy alone vs drainage	4 (23.5)
Cystectomy alone vs drainage + HS	1 (5.8)
Cystectomy + ST vs cystectomy + HS	1 (5.8)
Cystectomy + HS vs drainage	1 (5.8)
Cystectomy + HS vs drainage + HS	1 (5.8)
Drainage vs drainage + HS	1 (5.8)

**Risk of bias**	
Low risk	14 (82.3)
High risk	3 (17.6)

AMH, anti-Müllerian hormone; cystectomy + HS, cystectomy with hemostatic sealants; cystectomy + TXA i.v., cystectomy with tranexamic acid; cystectomy + ST, cystectomy with suture; drainage + HS, drainage with hemostatic sealants.

**Table 2.** Description of the available techniques for surgical treatment of endometrioma.

Technique	Way of performing	Disadvantages
Cystectomy	Excision of the endometrioma wall by gently pulling it from the underlying ovarian tissue. Hemostatis is usually performed via targeted coagulation on the ovarian tissue. Various variations can be performed before or after cystectomy, such as the use of hemostatic agents, tranexamic acid, and suturing of the ovary.	Often difficult to detach the endometrioma wall from the underlying ovarian tissue due to fibrosis so that the pulling of the capsule results in removing part of the underlying ovarian tissue and heavier bleeding.Subsequently, a more aggressive bipolar coagulation might be needed to achieve hemostasis, which further decreases the ovarian reserves.
Drainage	Fenestration of the endometrioma and aspiration of the endometrioma content. Targeted coagulation might be needed to achieve hemostasis.	High risk of endometrioma and symptom recurrence.
Drainage and use of hemostatic sealants	Fenestration of the endometrioma and aspiration of the endometrioma content followed by the use of hemostatic sealant (e.g. Surgicel), which is left in the ovary.	Not enough evidence regarding endometrioma and cyst recurrence.
Drainage and use of CO_2_ laser	Fenestration of the endometrioma and aspiration of the endometrioma content followed by laser ablation of the endometrioma wall. This can be performed either as a one-step procedure or three-step procedure (drainage, 3 months downregulation, laser ablation).	Might be time consuming for larger endometriomas and difficult to use in multiple endometriomas. Equipment cost.
Drainage and use of PlasmaJet	Fenestration of the endometrioma and aspiration of the endometrioma content followed by laser ablation of the endometrioma wall.	Similar to CO_2_ laser.
Alcohol sclerotherapy	Injection of the ethanol sclerosing agent into the cyst to destroy the endometrioma wall. It may potentially be performed not only laparoscopically but also transvaginally.	Not enough evidence regarding the effect of the ovarian reserves. Risk of cyst and symptom recurrence. No agreed concentration and duration of retention. If performed transvaginally, other endometriotic lesions in the abdomen cannot be removed.
Cystectomy with combined approach	Partial cystectomy is initially performed. When the excision causes bleeding or the cleavage plane is not clear, the rest of the cyst wall is vaporized using a CO_2_ laser	Not enough evidence regarding pain and fertility outcomes. Equipment cost.

The assessment of risk for bias (Supplementary Fig. S2) showed that five of the included studies had high risk of bias (Ghafarnejad *et al.*, 2014; Tanprasertkul *et al.*, 2014; Rouholamin and Ahmadpour-Ghazvini, 2019; Akkaranurakkul *et al.*, 2021, Ghasemi Tehrani *et al.*, 2022), while the rest included studies had low risk of bias.

### Primary outcome

**Figure 2.**
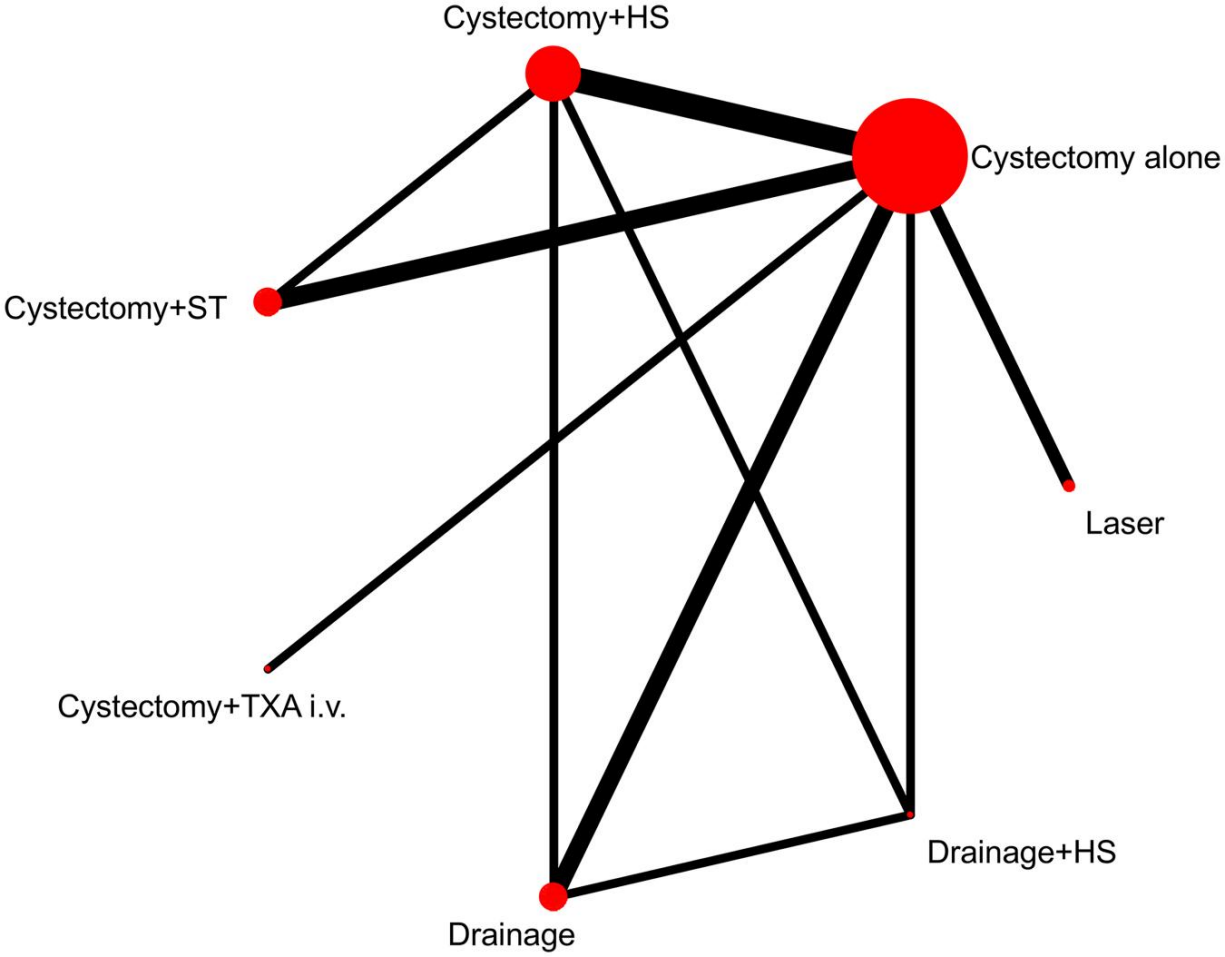
**Network plot of ‘AMH at endpoint (3–6 months)’.** The lines link interventions that were directly compared in trials. The thickness of the lines corresponds to the number of studies evaluating the comparison. The size of the nodes corresponds to the number of participants assigned to the intervention. AMH, anti-Müllerian hormone; cystectomy + HS, cystectomy with hemostatic sealants; cystectomy + TXA i.v., cystectomy with tranexamic acid; cystectomy + ST, cystectomy with suture; drainage + HS, drainage with hemostatic sealants.

**Figure 3.**
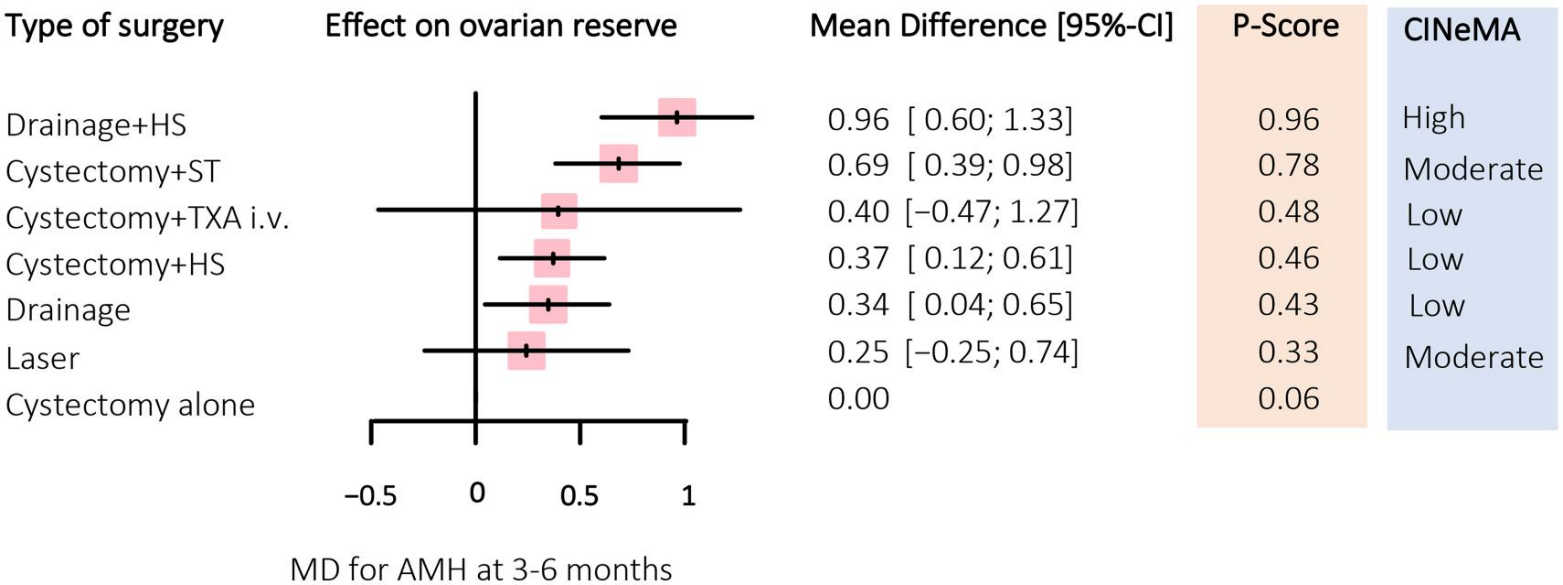
**Forest plot comparing each surgical technique with cystectomy alone for the on levels of AMH 3–6 months after surgery.** Confidence in Network Meta-Analysis (CINeMA) appraisal used for assessment of ranking probability and certainty of evidence. AMH, anti-Müllerian hormone; MD, mean difference; cystectomy + HS, cystectomy with hemostatic sealants; cystectomy + TXA i.v., cystectomy with tranexamic acid; cystectomy + ST, cystectomy with suture; drainage + HS, drainage with hemostatic sealants. *P*-scores (0–1) indicate the relative ranking of treatments, with higher values representing a higher effectiveness of the treatment.

**Figure 4.**
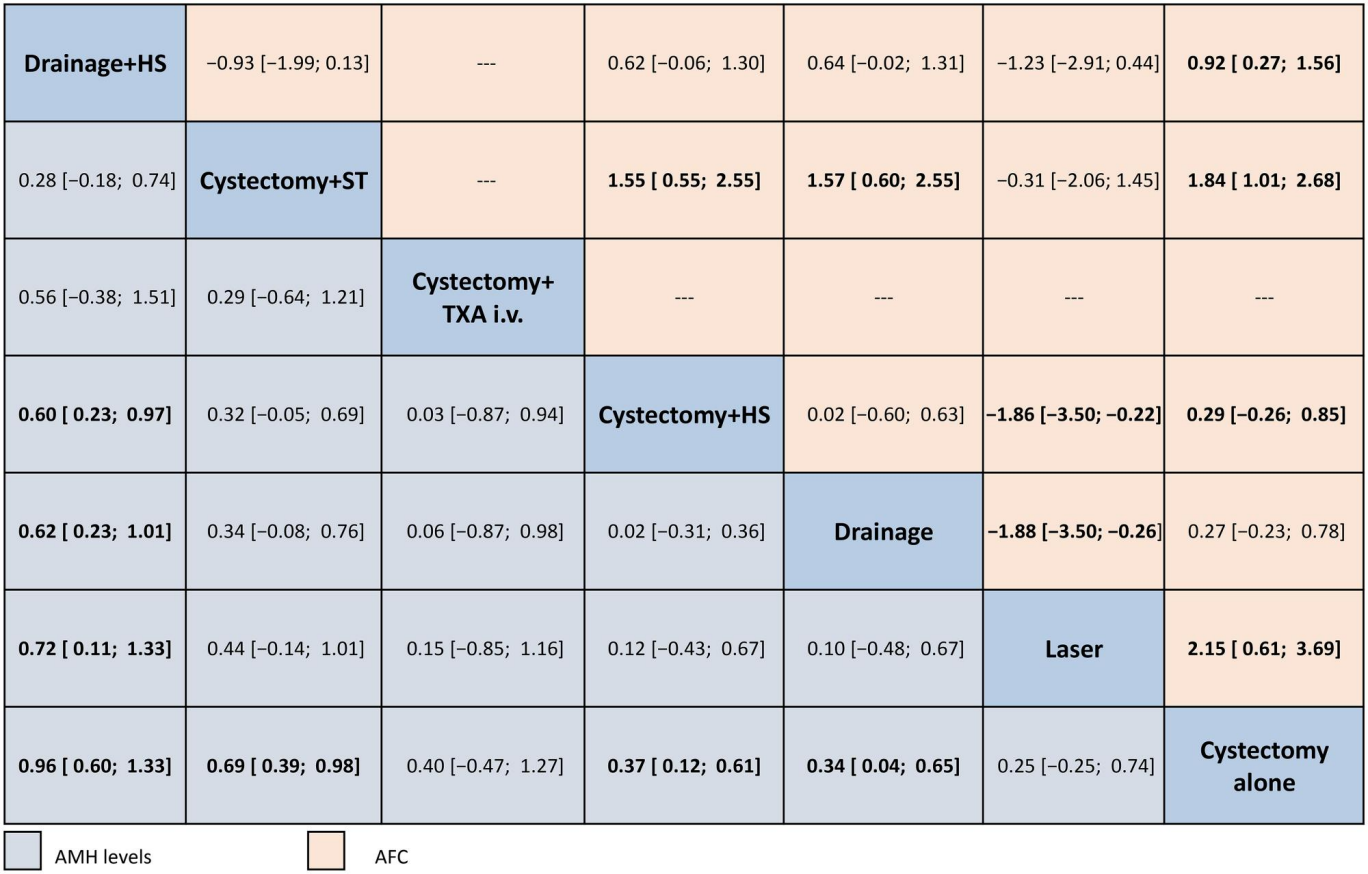
**Net league table of the head-to-head comparisons for the primary outcome of levels of AMH 3–6 months after surgery and the secondary outcome of AFC.** Outcomes are reported as mean differences (MDs) and 95% CIs. AMH results are shown in blue and AFC results in orange. AMH, anti-Müllerian hormone; AFC, antral follicle count; cystectomy + HS, cystectomy with hemostatic sealants; cystectomy + TXA i.v., cystectomy with tranexamic acid; cystectomy + ST, cystectomy with suture; drainage + HS, drainage with hemostatic sealants.

The network plot for the primary outcome is shown in Fig. 2. The results of the NMA for individual surgical treatments for the primary outcome are shown in Figs 3 and 4. Compared to the standard surgical approach, cystectomy alone, drainage + HS had the least detrimental effect on AMH, with an MD of 0.96 ng/ml (95% CI: 0.60–1.33; high certainty of evidence) followed by cystectomy + ST (MD: 0.69 ng/ml; 95% CI: 0.39–0.98; moderate certainty of evidence), cystectomy + HS (MD: 0.37 ng/ml; 95% CI: 0.12–0.61; low certainty of evidence), and drainage (MD: 0.34 ng/ml; 95% CI: 0.04–0.65; low certainty of evidence) (Fig. 3). Cystectomy + TXA i.v. and laser did not show any difference compared to the standard surgical approach, cystectomy alone (MD: 0.40 ng/ml; 95% CI: −0.47 to 1.27; low certainty of evidence and MD: 0.25 ng/ml; 95% CI: −0.25 to 0.74; moderate certainty of evidence, respectively). For the comparison of drainage + HS vs cystectomy, the certainty of evidence was high, as we did not find any evidence for downgrading the evaluation (i.e. no evidence of within the study bias, reporting bias, indirectness, imprecision, heterogeneity, or incoherence). Further details about the certainty of evidence according to the CINeMA assessment are presented in Supplementary Table S4.

Head-to-head comparison showed drainage + HS to be less detrimental than cystectomy + HS, drainage, laser, and cystectomy alone on ovarian reserve in terms of AMH levels. All the other comparisons showed no difference in their effects on AMH levels after surgery.

We found no evidence of significant inconsistency (Supplementary File S2).

The sensitivity analysis with only low risk of bias studies included 14 studies involving 7 surgical techniques (Tsolakidis *et al.*, 2010; Ferrero *et al.*, 2012; Sönmezer *et al.*, 2013; Giampaolino *et al.*, 2015; Asgari *et al.*, 2016; Zhang *et al.*, 2016; Candiani *et al.*, 2018; Choi *et al.*, 2018; Shaltout *et al.*, 2019; Sweed *et al.*, 2019; Javaheri *et al.*, 2021; Park *et al.*, 2021; Alborzi *et al.*, 2022; Araujo *et al.*, 2022). We found no substantial difference compared to the main analysis. Details are reported in Supplementary File S3.

The sensitivity analysis with only studies without imputations included 13 studies and 7 interventions, and the sensitivity analysis after exclusion of the studies with high baseline AMH included 13 studies and 7 interventions. We found for both analyses no substantial differences compared to the main analysis. Details are reported in Supplementary File S3.

### Subgroup analyses

The analysis considering AMH levels at two different time points, 3 and 6 months after surgery, included 13 (Ferrero *et al.*, 2012; Sönmezer *et al.*, 2013; Tanprasertkul *et al.*, 2014; Giampaolino *et al.*, 2015; Asgari *et al.*, 2016; Zhang *et al.*, 2016; Candiani *et al.*, 2018; Choi *et al.*, 2018; Chung *et al*., 2019; Akkaranurakkul *et al.*, 2021; Javaheri *et al.*, 2021; Park *et al.*, 2021; Alborzi *et al.*, 2022) and 8 trials, respectively (Tsolakidis *et al.*, 2010; Ferrero *et al.*, 2012; Tanprasertkul *et al.*, 2014; Zhang *et al.*, 2016; Rouholamin and Ahmadpour-Ghazvini, 2019; Shaltout *et al.*, 2019; Alborzi *et al.*, 2022; Araujo *et al.*, 2022) (Supplementary Table S5). Cystectomy + ST was the only intervention compared to cystectomy alone with higher AMH at three months after surgery (MD: 0.96 ng/ml; 95% CI: 0.64–1.28). The two interventions that showed significantly higher AMH levels 6 months after surgery in comparison to cystectomy were drainage + HS (MD: 1.00 ng/ml; 95% CI: 0.29–1.71) and cystectomy + ST (MD: 0.52 ng/ml; 95% CI: 0.25–1.17). Two more subgroup analyses were performed according to the localization of the endometrioma (unilateral and bilateral) and the size of the endometrioma (< or ≥ 5 cm) (Supplementary Table S5). The subgroup analysis of studies with only unilateral endometriomas (N = 10) showed similar results to the main analysis. When studies with both unilateral and bilateral endometriomas were included (N = 6), no statistically significant difference was found between the interventions and cystectomy alone. Concerning the endometrioma size, only three studies included endometriomas with size <5 cm, and no difference among the interventions was found. For endometriomas ≥5 cm, the results were in concordance with the results of the main analysis.

### Secondary outcomes

Regarding the reduction of AFC levels at 3–6 months after surgery (Supplementary File S4), compared to cystectomy alone, laser showed the lowest reduction of AFC (MD: 2.30; 95% CI: 0.20–4.40), followed by cystectomy + ST (MD: 1.88; 95% CI: 0.98–2.79) and drainage + HS (MD: 0.92; 95% CI: 0.19–1.65). In the head-to-head comparisons, cystectomy + ST resulted in significantly lower AFC reduction compared to cystectomy + HS (MD: 1.58; 95% CI: 0.49–2.67) and drainage (MD: 1.61; 95% CI: 0.55–2.68) (Supplementary File S4).

In terms of the recurrence rate, nine studies reported the outcome. We observed significant variability among the included studies. In studies involving drainage, the recurrence rate varied from 27% (Shaltout *et al.*, 2019) to 41.5% (Sweed *et al.*, 2019) for follow-up between 12 and 24 months. Shaltout *et al.* (2019) observed a lower recurrence rate after drainage with HS (10.8%) at 24 months. Regarding cystectomy alone, the recurrence rate ranges from 0 cases for a follow-up between 3 and 12 months (Tsolakidis *et al.*, 2010; Candiani *et al.*, 2018; Park *et al.*, 2021) to 24.4% after a 24-month follow-up (Shaltout *et al.*, 2019). The recurrence rates for variations of cystectomy, such as HS, varied between 0% (Park *et al.*, 2021) after 3 months of follow-up and 9% after 24 months of follow-up (Shaltout *et al.*, 2019). For cystectomy with sutures, the recurrence rate ranged from 0% (Zhang *et al.*, 2016) to 8.8% (Ferrero *et al.*, 2012) after 12 months of follow-up. Recurrence after laser treatment of ovarian endometrioma ranged from 0% (Candiani *et al.*, 2018) after a mean follow-up of 8.1 months to 20% after a 12-month follow-up (Tsolakidis *et al.*, 2010), and finally sclerotherapy showed a recurrence rate of 48.5% after 12 months of follow-up (Ghasemi Tehrani *et al.*, 2022).

Concerning pregnancy outcomes, as the majority of the included studies had a follow-up of up to 6 months, only four studies with longer follow-up reported pregnancy rates (Ferrero *et al.*, 2012; Candiani *et al.*, 2018; Shaltout *et al.*, 2019; Alborzi *et al.*, 2022). Shaltout *et al.* (2019) had only spontaneous pregnancies, while Alborzi *et al.* (2022) had both spontaneous and pregnancies after ART. Pregnancy rates after cystectomy + HS varied from 13.6% after 24 months of follow-up (Shaltout *et al.*, 2019) to 40% after 12 months of follow-up (Alborzi *et al.*, 2022). Meanwhile, 12 months after cystectomy + ST, Ferrero *et al.* (2012) found a 44.1% pregnancy rate; however, the author did not report if the pregnancies were spontaneous or after ART. Candiani *et al.* (2018) reported a 10% spontaneous pregnancy rate after laser treatment for a mean follow-up time of 8.1 months.

## Discussion

To the best of our knowledge, this is the first NMA comparing the impact of different endometrioma surgical treatments on the ovarian reserves (Supplementary File S5).

Our results indicate that both drainage combined with the use of HS and cystectomy with ST may have a less detrimental impact on AMH levels compared to cystectomy alone, measured 3–6 months post-surgery. This conclusion is supported by high and moderate certainty of evidence, respectively, based on the CINeMA assessment. Sensitivity analyses restricting to high-quality studies, excluding those with data imputations, and separately examining AMH outcomes at 3 and 6 months consistently confirmed these findings. Additionally, the results remained robust when excluding two studies with unusually high preoperative AMH levels. However, in the case of small endometriomas (<5 cm), no significant differences were observed, though this conclusion is limited by the inclusion of only three studies in the meta-analysis.

Although the impact of drainage with HS and cystectomy with ST on AMH levels may appear modest compared to cystectomy alone—with differences ranging from 0.70 to 1 ng/ml—AMH serves as an important indicator of surgical damage to the ovaries. Even a slight advantage in preserving AMH levels with one technique over another could be clinically significant, particularly for patients with already diminished ovarian reserve, and thus valuable information for clinicians when selecting the optimal surgical approach. However, further research is necessary to understand whether the benefits of HS or ST outweigh the possible risks related to their use, such as recurrence or inflammation and fibrosis.

The current meta-analysis showed no difference between laser and cystectomy in terms of AMH. It should be noted, however, that other single studies (Donnez *et al.*, 1996; Candiani *et al.*, 2018) have demonstrated that ablative techniques with CO_2_ laser had less impact on the ovarian reserve than cystectomy.

Regarding the AFC levels at 3–6 months after surgery, laser showed better results followed by cystectomy + ST and drainage + HS. Again, the beneficial effect, between 0.9 and 2.30 antral follicles, could be of clinical relevance. It is important that both the three-step laser procedure using GnRH agonists for 3 months between drainage and CO_2_ laser ablation (Tsolakidis *et al*, 2010) and the one-step laser procedure (Candiani *et al.*, 2018) found a similar positive effect on AFC compared to simple cystectomy. The former technique has been inspired by Donnez *et al.* (1996), who published the largest series 30 years ago, but it is currently infrequently applied due to the need for multiple procedures and complexity.

A possible explanation of the above findings is the different amount of energy (bipolar or monopolar coagulation) applied to the remaining ovarian tissue after cystectomy to achieve hemostasis. Suture of the ovarian cortex and HS such as cellulose or fibrinogen and thrombin-containing materials possibly decrease the need for coagulation on the remaining ovarian tissue and therefore may decrease the coagulation-associated ovarian damage (Shaltout *et al.*, 2019). Moreover, for drainage with HS as well as for laser ablation, no detaching of the cyst is performed, therefore limiting the accidental removal of healthy tissue with the endometrioma wall (Tulikangas *et al.*, 2001).

A consensus study published by an international working group of the European Society for Gynecologic Endoscopy (ESGE), ESHRE, and the World Endometriosis Society (WES) suggested cystectomy, ablation by laser or plasma energy, and electrocoagulation, or a combination of them, for the endometrioma removal. Injection of diluted vasopressin solution under the cyst capsule, as well as ST or the use of intraovarian HS agents, were also recommended (Saridogan *et al.*, 2017). Due to the scarcity of evidence, however, recommendations were mainly based on expert opinion on best clinical practice.

The long-term impact of cystectomy for endometrioma on ovarian reserve remains controversial. Normal folliculogenesis takes ∼3 months; therefore, in theory, a stabilization of AMH would be expected after that period postoperatively. However, several observational studies have suggested a significant recovery of AMH during 1-year post-surgical follow-up (Sugita *et al.*, 2013; Vignali *et al.*, 2015; Kovačević *et al.*, 2018; Kostrzewa *et al.*, 2019; Wang *et al.*, 2019). The studies included in our meta-analysis mainly provided AMH values up to 6 months post-surgery, limiting our ability to draw conclusions on longer-term outcomes or provide recommendations on when to initiate ovarian stimulation for ART if postoperatively needed.

A systematic review and meta-analysis of observational and randomized studies analyzing pre- and post-surgical AFC levels showed that ovarian reserve was not reduced 3–6 months after endometrioma cystectomy (Muzii *et al.*, 2014). In contrast, another meta-analysis reported diminished ovarian reserves measured by AMH (Raffi *et al.*, 2012), indicating a reduction in ovarian reserve after all types of surgical treatments. These discrepancies may be attributed to differences in the outcome measurement (AFC vs AMH) and the heterogeneity of studies included. Moreover, AFC might be improper for estimating the ovarian reserve in ovaries with endometriomas. The presence of a large endometrioma may impair the sonographic identification of small follicles adjacent to the cyst, and, consequently, ovarian reserve could be underestimated before surgery.

Regarding the recurrence of endometrioma, included studies showed significant variability, with the recurrence rate ranging from 0% after cystectomy to 41.5% one year after endometrioma drainage. Previous randomized controlled studies have shown that endometrioma recurrence tends to be high after endometrioma drainage (Beretta *et al.*, 1998; Alborzi *et al.*, 2022), likely due to the incomplete removal of endometriotic tissue. Interestingly, however, one study reported a lower recurrence rate (10.9%) at 24 months when oxidized cellulose was used during drainage, comparable to traditional cystectomy outcomes (Shaltout *et al.*, 2019). The authors suggested that oxidized regenerated cellulose may exert a chemical ablation effect on the ectopic endometriotic tissue by creating a highly acidic environment (pH 2–4) and inducing severe vasoconstriction, leading to tissue anoxia and potentially eliminating residual endometrial cells. Further research is needed to validate these findings. In terms of laser ablation, a recent study with a 3-year follow-up showed that CO_2_ laser ablation had similar a recurrence risk compared to cystectomy (4.9% vs 6.3%) (Candiani *et al.*, 2018). It is important to note that postoperative adjuvant hormonal treatment remains a crucial protective factor against endometrioma recurrence (Vercellini *et al.*, 2013) and should be considered regardless of the surgical technique employed.

Adjuvant treatments play an important role in preventing endometrioma recurrence, but impact AMH and AFC as well. Women who had used combined oral contraceptive pills (COC) for more than a year had significantly less AFC as well as a smaller ovarian volume compared with the control group (Deb *et al.*, 2012). Regarding the effect of hormonal contraceptives on AMH, a cross-sectional cohort study including 27 125 women compared serum levels of AMH in contraceptive and non-contraceptive users (Hariton *et al.*, 2021). The results showed that AMH levels were significantly lower in women using COC, vaginal ring, hormonal intrauterine device, implant, or progestin-only pill than in non-contraceptive users. None of the studies included in our meta-analysis reported the use of postoperative hormonal treatment, minimizing the risk of bias from this factor in our results.

Several other factors can contribute to over- or underscoring of AMH and AFC measurements. Manual assays for AMH have been prone to variability, but the development of fully automated assays has improved consistency. Moreover, although less significant with automated systems, operator skill can still influence results if manual steps are involved. On the other hand, AFC is highly dependent on the skill of the operator performing ultrasound scans, leading to significant inter-operator variability. Again, the inclusion of only randomized controlled studies should have balanced these risks between study arms and represents, therefore, the strength of our study.

Regarding pregnancy outcomes, most of the included studies did not report pregnancy rates, despite their clinical relevance and impact on quality of life, underscoring a critical gap in the research. As a result, we were unable to conduct any further analysis on this outcome and strongly recommend that future studies prioritize the inclusion of clinically relevant outcomes, such as pregnancy rates. A multicentric non-randomized comparative study found that pregnancy rates were similar after treating ovarian endometrioma with either plasma energy ablation or cystectomy (Mircea *et al.*, 2016). Additionally, a meta-analysis of seven RCTs showed that cystectomy was associated with higher conception rates compared to drainage, though not when compared to laser ablation (Dan and Limin, 2013).

The results of this work need to be interpreted with caution due to a number of limitations. Several sources of heterogeneity may reduce the certainty of evidence. There was heterogeneity regarding the time of AMH measurement, ranging from 3 to 6 months, which could have an impact on the value found, as AMH levels might need some time to increase back to normality after surgery. This could have resulted in an underestimation of AMH levels after surgery. To mitigate this potential bias, we explored potential differences in terms of the timing of AMH measurement in a subgroup analysis. Additionally, it could be argued that long-term rather than short-term AMH values are more relevant indicators of the ovarian reserve. However, only a few studies measured AMH at 12 months; therefore, we could not perform any analysis on the long-term effects of surgery on the ovarian reserve. Although we did not find any evidence of significant inconsistency, it is possible that some residual heterogeneity and inconsistency exist. Additionally, for certain outcomes such as pregnancy rates, the generally short follow-up durations limited data availability, precluding planned analyses and restricting conclusions regarding long-term fertility outcomes. Third, the included RCTs were heterogeneous in terms of localization and size of endometrioma, which we tried to explore with two additional subgroup analyses. Fourth, different surgical expertise might have an intrinsic effect on the result of the surgery. Fifth, sclerotherapy, a technique for endometrioma surgery that has become more popular in recent years, was not included in the NMA because the only study found reported discrepant outcomes, and the corresponding author did not respond to our emails. Finally, although the mean age, BMI, and endometrioma size in our study are representative and generalizable to other patients with endometriosis, the mean AMH of 3.58 ng/ml before surgery reflects patients with generally good ovarian reserves; therefore our findings should be interpreted with caution for patients with low ovarian reserve.

### Clinical and research implications

The findings of this study suggest that laparoscopic cystectomy with ovarian ST as well as endometrioma drainage combined with the application of HS are associated with smaller short-term declines in ovarian reserve markers compared to traditional laparoscopic cystectomy. However, the clinical relevance of these differences is uncertain, given the modest effect sizes and reliance on surrogate outcomes. Potential risks, including inflammation and fibrosis from allogenic materials applied to the ovary, warrant careful consideration. Moreover, the simple drainage technique using HS (oxidative regenerated cellulose) was evaluated in only one study, and potential bias due to the specific setting and surgical expertise cannot be ruled out. Further research, including well-powered trials assessing fertility and long-term outcomes, is needed to clarify the safety and efficacy of these techniques.

Future research should also explore the potential of plasma energy or sclerotherapy, as there is currently a lack of RCT data on these procedures. Additionally, CO_2_ laser ablation appears to be less harmful to ovarian reserve, as measured by AFC in the current study and AMH in several other studies (Donnez *et al.*, 1996; Candiani *et al.*, 2018) and may be preferred over simple cystectomy, although it is typically associated with higher costs and technical challenges.

This review excluded non-randomized studies, such as large prospective cohorts, to reduce the risk of bias and ensure the highest level of evidence (Murad *et al.*, 2016). We recognize that this limited the number of included studies and patients, and future well-designed randomized trials are needed to fill this gap.

Given the widespread prevalence of endometrioma surgery, there is an urgent need for adequately powered, RCTs conducted through collaborative networks involving endometriosis societies and multiple specialized centers. These studies should follow a standardized protocol, specifying precise timing and methodologies for measuring AMH and AFC, alongside a comprehensive evaluation of reproductive outcomes. An extended follow-up duration is essential to obtain robust longitudinal data and to adequately assess long-term outcomes. Additionally, surgical techniques must be clearly defined and consistently performed by designated surgeons within each center to standardize surgical expertise and minimize variability in outcomes. Importantly, since endometriosis lesions outside the ovaries can impair natural conception, surgical approaches should not only target endometriomas but also aim to restore pelvic anatomy and excise all visible endometriosis lesions.

## Supplementary Material

hoag019_Supplementary_Data

## Data Availability

The data that support the findings of this study are available from the corresponding author upon reasonable request.

## References

[hoag019-B1] Akkaranurakkul P , Lertvikool S, Hongsakorn W, Vallibhakara O, Tantanavipas S, Paiwattananupant K, Ittichaikulthol W, Vongsakulyanon A, Vallibhakara SA, Anantaburana M et al Effects of intravenous tranexamic acid on ovarian reserve and intra-operative blood loss during laparoscopic cystectomy of endometriotic cyst: a pilot randomized controlled trial. Pilot Feasibility Stud 2021;7:171. 34481524 10.1186/s40814-021-00907-y PMC8417623

[hoag019-B2] Alborzi S , Poordast T, Askary E, Chamanara K, Zahiri Sorouri Z, Hosseini Najar Kellaii E, Pirzadeh Nahooji S. The effect of vasopressin injection on ovarian reserve in patients with ovarian endometrioma: a randomized controlled trial. Reprod Biomed Online 2022;44:651–658. 35272940 10.1016/j.rbmo.2021.11.024

[hoag019-B3] Araujo R , Maia SB, Baracat CMF, Fernandes C, Ribeiro H, Ribeiro P. Ovarian function following use of various hemostatic techniques during treatment for unilateral endometrioma: a randomized controlled trial. Int J Gynaecol Obstet 2022;157:549–556. 34478564 10.1002/ijgo.13912

[hoag019-B4] Asgari Z , Rouholamin S, Hosseini R, Sepidarkish M, Hafizi L, Javaheri A. Comparing ovarian reserve after laparoscopic excision of endometriotic cysts and hemostasis achieved either by bipolar coagulation or suturing: a randomized clinical trial. Arch Gynecol Obstet 2016;293:1015–1022. 26493551 10.1007/s00404-015-3918-4

[hoag019-B5] Becker CM , Bokor A, Heikinheimo O, Horne A, Jansen F, Kiesel L, King K, Kvaskoff M, Nap A, Petersen K et al; ESHRE Endometriosis Guideline Group. ESHRE guideline: endometriosis. Hum Reprod Open 2022;2022:hoac009. 35350465 10.1093/hropen/hoac009 PMC8951218

[hoag019-B6] Beretta P , Franchi M, Ghezzi F, Busacca M, Zupi E, Bolis P. Randomized clinical trial of two laparoscopic treatments of endometriomas: cystectomy versus drainage and coagulation. Fertil Steril 1998;70:1176–1180. 9848316 10.1016/s0015-0282(98)00385-9

[hoag019-B7] Bulun SE. Endometriosis. N Engl J Med 2009;360:268–279. 19144942 10.1056/NEJMra0804690

[hoag019-B8] Candiani M , Ottolina J, Posadzka E, Ferrari S, Castellano LM, Tandoi I, Pagliardini L, Nocun A, Jach R. Assessment of ovarian reserve after cystectomy versus 'one-step’ laser vaporization in the treatment of ovarian endometrioma: a small randomized clinical trial. Hum Reprod 2018;33:2205–2211. 30299482 10.1093/humrep/dey305 PMC6238368

[hoag019-B9] Carmona F , Martínez-Zamora MA, Rabanal A, Martínez-Román S, Balasch J. Ovarian cystectomy versus laser vaporization in the treatment of ovarian endometriomas: a randomized clinical trial with a five-year follow-up. Fertil Steril 2011;96:251–254. 21575941 10.1016/j.fertnstert.2011.04.068

[hoag019-B10] Choi C , Kim WY, Lee DH, Lee SH. Usefulness of hemostatic sealants for minimizing ovarian damage during laparoscopic cystectomy for endometriosis. J Obstet Gynaecol Res 2018;44:532–539. 29271052 10.1111/jog.13542

[hoag019-B11] Chung J , Law T, Chung C, Mak J, Sahota DS, Li TC. Impact of haemostatic sealant versus electrocoagulation on ovarian reserve after laparoscopic ovarian cystectomy of ovarian endometriomas: a randomised controlled trial. BJOG 2019;126:1267–1275. 31038276 10.1111/1471-0528.15807

[hoag019-B12] Cohen A , Almog B, Tulandi T. Sclerotherapy in the management of ovarian endometrioma: systematic review and meta-analysis. Fertil Steril 2017;108:117–124.e5. 28579409 10.1016/j.fertnstert.2017.05.015

[hoag019-B13] Coric M , Barisic D, Pavicic D, Karadza M, Banovic M. Electrocoagulation versus suture after laparoscopic stripping of ovarian endometriomas assessed by antral follicle count: preliminary results of randomized clinical trial. Arch Gynecol Obstet 2011;283:373–378. 20844886 10.1007/s00404-010-1676-x

[hoag019-B14] Dan H , Limin F. Laparoscopic ovarian cystectomy versus fenestration/coagulation or laser vaporization for the treatment of endometriomas: a meta-analysis of randomized controlled trials. Gynecol Obstet Invest 2013;76:75–82. 23751250 10.1159/000351165

[hoag019-B15] Deb S , Campbell BK, Pincott-Allen C, Clewes JS, Cumberpatch G, Raine-Fenning NJ. Quantifying effect of combined oral contraceptive pill on functional ovarian reserve as measured by serum anti-Müllerian hormone and small antral follicle count using three-dimensional ultrasound. Ultrasound Obstet Gynecol 2012;39:574–580. 21997961 10.1002/uog.10114

[hoag019-B16] Donnez J , Nisolle M, Gillet N, Smets M, Bassil S, Casanas-Roux F. Large ovarian endometriomas. Hum Reprod 1996;11:641–646. 8671283 10.1093/humrep/11.3.641

[hoag019-B17] Ferrero S , Venturini PL, Gillott DJ, Remorgida V, Leone Roberti Maggiore U. Hemostasis by bipolar coagulation versus suture after surgical stripping of bilateral ovarian endometriomas: a randomized controlled trial. J Minim Invasive Gynecol 2012;19:722–730. 23084676 10.1016/j.jmig.2012.08.001

[hoag019-B18] Ghafarnejad M , Akrami M, Davari-Tanha F, Adabi K, Nekuie S. Vasopressin effect on operation time and frequency of electrocauterization during laparoscopic stripping of ovarian endometriomas: a randomized controlled clinical trial. J Reprod Infertil 2014;15:199–204. 25473628 PMC4227977

[hoag019-B19] Ghasemi Tehrani H , Tavakoli R, Hashemi M, Haghighat S. Ethanol sclerotherapy versus laparoscopic surgery in management of ovarian endometrioma; a randomized clinical trial. Arch Acad Emerg Med 2022;10:e55. 36033993 10.22037/aaem.v10i1.1636 PMC9397592

[hoag019-B20] Giampaolino P , Bifulco G, Di Spiezio Sardo A, Mercorio A, Bruzzese D, Di Carlo C. Endometrioma size is a relevant factor in selection of the most appropriate surgical technique: a prospective randomized preliminary study. Eur J Obstet Gynecol Reprod Biol 2015;195:88–93. 26492167 10.1016/j.ejogrb.2015.09.046

[hoag019-B21] Gylfason JT , Kristjansson KA, Sverrisdottir G, Jonsdottir K, Rafnsson V, Geirsson RT. Pelvic endometriosis diagnosed in an entire nation over 20 years. Am J Epidemiol 2010;172:237–243. 20616202 10.1093/aje/kwq143

[hoag019-B22] Hariton E , Shirazi TN, Douglas NC, Hershlag A, Briggs SF. Anti-Müllerian hormone levels among contraceptive users: evidence from a cross-sectional cohort of 27,125 individuals. Am J Obstet Gynecol 2021;225:515.e511–515.e510. 10.1016/j.ajog.2021.06.052 34126087

[hoag019-B24] Javaheri A , Ashkezar SK, Eftekhar M, Tafti SZG. Ovarian reserve in women with endometriosis under total cystectomy compared to partial cystectomy: a randomized clinical trial. Int J Reprod Biomed 2021;19:619–624. 34458670 10.18502/ijrm.v19i7.9472 PMC8387706

[hoag019-B25] Kalaitzopoulos DR , Samartzis N, Kolovos GN, Mareti E, Samartzis EP, Eberhard M, Dinas K, Daniilidis A. Treatment of endometriosis: a review with comparison of 8 guidelines. BMC Womens Health 2021;21:397. 34844587 10.1186/s12905-021-01545-5 PMC8628449

[hoag019-B26] Kostrzewa M , Wilczyński JR, Głowacka E, Żyła M, Szyłło K, Stachowiak G. One-year follow-up of ovarian reserve by three methods in women after laparoscopic cystectomy for endometrioma and benign ovarian cysts. Int J Gynaecol Obstet 2019;146:350–356. 31197834 10.1002/ijgo.12884

[hoag019-B27] Kovačević VM , Anđelić LM, Mitrović Jovanović A. Changes in serum antimüllerian hormone levels in patients 6 and 12 months after endometrioma stripping surgery. Fertil Steril 2018;110:1173–1180. 30396562 10.1016/j.fertnstert.2018.07.019

[hoag019-B28] Liu X , Yuan L, Shen F, Zhu Z, Jiang H, Guo SW. Patterns of and risk factors for recurrence in women with ovarian endometriomas. Obstet Gynecol 2007;109:1411–1420. 17540815 10.1097/01.AOG.0000265215.87717.8b

[hoag019-B29] Luo D , Wan X, Liu J, Tong T. Optimally estimating the sample mean from the sample size, median, mid-range, and/or mid-quartile range. Stat Methods Med Res 2018;27:1785–1805. 27683581 10.1177/0962280216669183

[hoag019-B30] Mbuagbaw L , Rochwerg B, Jaeschke R, Heels-Andsell D, Alhazzani W, Thabane L, Guyatt GH. Approaches to interpreting and choosing the best treatments in network meta-analyses. Syst Rev 2017;6:79. 28403893 10.1186/s13643-017-0473-z PMC5389085

[hoag019-B31] Mircea O , Puscasiu L, Resch B, Lucas J, Collinet P, von Theobald P, Merviel P, Roman H. Fertility outcomes after ablation using plasma energy versus cystectomy in infertile women with ovarian endometrioma: a multicentric comparative study. J Minim Invasive Gynecol 2016;23:1138–1145. 27553184 10.1016/j.jmig.2016.08.818

[hoag019-B32] Murad MH , Asi N, Alsawas M, Alahdab F. New evidence pyramid. Evid Based Med 2016;21:125–127. 27339128 10.1136/ebmed-2016-110401 PMC4975798

[hoag019-B33] Muzii L , Di Tucci C, Di Feliciantonio M, Galati G, Di Donato V, Musella A, Palaia I, Panici PB. Antimüllerian hormone is reduced in the presence of ovarian endometriomas: a systematic review and meta-analysis. Fertil Steril 2018;110:932–940.e931. 30316440 10.1016/j.fertnstert.2018.06.025

[hoag019-B34] Muzii L , Di Tucci C, Di Feliciantonio M, Marchetti C, Perniola G, Panici PB. The effect of surgery for endometrioma on ovarian reserve evaluated by antral follicle count: a systematic review and meta-analysis. Hum Reprod 2014;29:2190–2198. 25085800 10.1093/humrep/deu199

[hoag019-B35] Park SJ , Seol A, Lee N, Lee S, Kim HS, Seol A, Lee E, Yim GW, Paik H, Kim HS et al; PRAHA Study Group. A randomized controlled trial of ovarian reserve preservation and hemostasis during ovarian cystectomy. Sci Rep 2021;11:8495. 33875738 10.1038/s41598-021-87965-7 PMC8055671

[hoag019-B36] Porpora MG , Pallante D, Ferro A, Crisafi B, Bellati F, Benedetti Panici P. Pain and ovarian endometrioma recurrence after laparoscopic treatment of endometriosis: a long-term prospective study. Fertil Steril 2010;93:716–721. 19061997 10.1016/j.fertnstert.2008.10.018

[hoag019-B37] Raffi F , Metwally M, Amer S. The impact of excision of ovarian endometrioma on ovarian reserve: a systematic review and meta-analysis. J Clin Endocrinol Metab 2012;97:3146–3154. 22723324 10.1210/jc.2012-1558

[hoag019-B38] Rouholamin S , Ahmadpour-Ghazvini F. Evaluation of ovarian reserve after laparoscopic cystectomy for endometrioma with or without vasopressin injection: a randomized clinical trial study. J Isfahan Med Sch 2019;37:48–54.

[hoag019-B39] Saridogan E , Becker CM, Feki A, Grimbizis GF, Hummelshoj L, Keckstein J, Nisolle M, Tanos V, Ulrich UA, Vermeulen N et al; Working group of ESGE, ESHRE, and WES. Recommendations for the surgical treatment of endometriosis-part 1: ovarian endometrioma. Gynecol Surg 2017;14:27. 29285022 10.1186/s10397-017-1029-x PMC5735196

[hoag019-B40] Shaltout MF , Elsheikhah A, Maged AM, Elsherbini MM, Zaki SS, Dahab S, Elkomy RO. A randomized controlled trial of a new technique for laparoscopic management of ovarian endometriosis preventing recurrence and keeping ovarian reserve. J Ovarian Res 2019;12:66. 31325962 10.1186/s13048-019-0542-0 PMC6642736

[hoag019-B42] Sönmezer M , Taşkın S, Gemici A, Kahraman K, Özmen B, Berker B, Atabekoğlu C. Can ovarian damage be reduced using hemostatic matrix during laparoscopic endometrioma surgery? A prospective, randomized study. Arch Gynecol Obstet 2013;287:1251–1257. 23291972 10.1007/s00404-012-2704-9

[hoag019-B43] Sterne JAC , Savović J, Page MJ, Elbers RG, Blencowe NS, Boutron I, Cates CJ, Cheng H-Y, Corbett MS, Eldridge SM et al RoB 2: a revised tool for assessing risk of bias in randomised trials. BMJ 2019;366:l4898. 31462531 10.1136/bmj.l4898

[hoag019-B44] Sugita A , Iwase A, Goto M, Nakahara T, Nakamura T, Kondo M, Osuka S, Mori M, Saito A, Kikkawa F. One-year follow-up of serum antimüllerian hormone levels in patients with cystectomy: are different sequential changes due to different mechanisms causing damage to the ovarian reserve? Fertil Steril 2013;100:516–522.e3. 23579006 10.1016/j.fertnstert.2013.03.032

[hoag019-B45] Sweed MS , Makled AK, El-Sayed MA, Shawky ME, Abd-Elhady HA, Mansour AM, Mohamed RM, Hemeda H, Nasr-Eldin EA, Attia NS et al Ovarian reserve following laparoscopic ovarian cystectomy vs cyst deroofing for endometriomas. J Minim Invasive Gynecol 2019;26:877–882. 30193971 10.1016/j.jmig.2018.06.022

[hoag019-B46] Tanprasertkul C , Ekarattanawong S, Sreshthaputra O, Vutyavanich T. Impact of hemostasis methods, electrocoagulation versus suture, in laparoscopic endometriotic cystectomy on the ovarian reserve: a randomized controlled trial. J Med Assoc Thai 2014;97:S95–S101. 25518300

[hoag019-B47] Tsolakidis D , Pados G, Vavilis D, Athanatos D, Tsalikis T, Giannakou A, Tarlatzis BC. The impact on ovarian reserve after laparoscopic ovarian cystectomy versus three-stage management in patients with endometriomas: a prospective randomized study. Fertil Steril 2010;94:71–77. 19393996 10.1016/j.fertnstert.2009.01.138

[hoag019-B48] Tulikangas PK , Smith T, Falcone T, Boparai N, Walters MD. Gross and histologic characteristics of laparoscopic injuries with four different energy sources. Fertil Steril 2001;75:806–810. 11287039 10.1016/s0015-0282(00)01785-4

[hoag019-B49] Vercellini P , DE Matteis S, Somigliana E, Buggio L, Frattaruolo MP, Fedele L. Long-term adjuvant therapy for the prevention of postoperative endometrioma recurrence: a systematic review and meta-analysis. Acta Obstet Gynecol Scand 2013;92:8–16. 22646295 10.1111/j.1600-0412.2012.01470.x

[hoag019-B50] Vignali M , Mabrouk M, Ciocca E, Alabiso G, Barbasetti di Prun A, Gentilini D, Busacca M. Surgical excision of ovarian endometriomas: does it truly impair ovarian reserve? Long term anti-Müllerian hormone (AMH) changes after surgery. J Obstet Gynaecol Res 2015;41:1773–1778. 26420658 10.1111/jog.12830

[hoag019-B51] Wan X , Wang W, Liu J, Tong T. Estimating the sample mean and standard deviation from the sample size, median, range and/or interquartile range. BMC Med Res Methodol 2014;14:135. 25524443 10.1186/1471-2288-14-135 PMC4383202

[hoag019-B52] Wang Y , Ruan X, Lu D, Sheng J, Mueck AO. Effect of laparoscopic endometrioma cystectomy on anti-Müllerian hormone (AMH) levels. Gynecol Endocrinol 2019;35:494–497. 30732484 10.1080/09513590.2018.1549220

[hoag019-B53] Younis JS , Shapso N, Ben-Sira Y, Nelson SM, Izhaki I. Endometrioma surgery—a systematic review and meta-analysis of the effect on antral follicle count and anti-Müllerian hormone. Am J Obstet Gynecol 2022;226:33–51.e37. 34265271 10.1016/j.ajog.2021.06.102

[hoag019-B54] Zhang CH , Wu L, Li PQ. Clinical study of the impact on ovarian reserve by different hemostasis methods in laparoscopic cystectomy for ovarian endometrioma. Taiwan J Obstet Gynecol 2016;55:507–511. 27590372 10.1016/j.tjog.2015.08.026

[hoag019-B55] Zondervan KT , Becker CM, Missmer SA. Endometriosis. N Engl J Med 2020;382:1244–1256. 32212520 10.1056/NEJMra1810764

