# Supplementary material for: Comparative effect of different surgical treatments for ovarian endometrioma on anti-Müllerian hormone levels: a systematic review and network meta-analysis
Source: Hum Reprod Open. 2026 Mar 1;2026(3):hoag019. doi: 10.1093/hropen/hoag019 (PMC13186601; doi:10.1093/hropen/hoag019)
Supplement: hoag019_Supplementary_Data [file hoag019_supplementary_data.docx]

Supplementary material

**Comparative effect of different surgical treatments for ovarian endometrioma on AMH levels: a systematic review and network meta-analysis**

Konstantinos Nirgianakis, Dimitrios Rafail Kalaitzopoulos, Nikolaus Fadinger, Michael D Mueller, Chiara Gastaldon

Contents

# **Supplementary Figure S1.** Transitivity check

# **Supplementary Figure S2.** Risk of bias assessment using revised Cochrane risk-of-bias tool for randomized trials (RoB 2)

**Supplementary Table S1.** List of excluded studies with reasons

**Supplementary Table S2.** Characteristics of the included studies

**Supplementary Table S3.** Description of the interventions framed following the network nodes classification

**Supplementary Table S4.** Certainty of evidence according to CiNEMA assessment

**Supplementary Table S5.** Subgroup analyses

**Supplementary File S1.** Search strategy

# **Supplementary File S2.** Additional results of the network meta-analysis of the primary outcome: AMH at endpoint (3-6 months)

# **Supplementary File S3.** Sensitivity analyses

# **Supplementary File S4.** Network plot of secondary outcome “AFC at endpoint (3-6 months)

# **Supplementary File S5.** Differences between protocol and review

# **Supplementary Figure S1.** Transitivity check


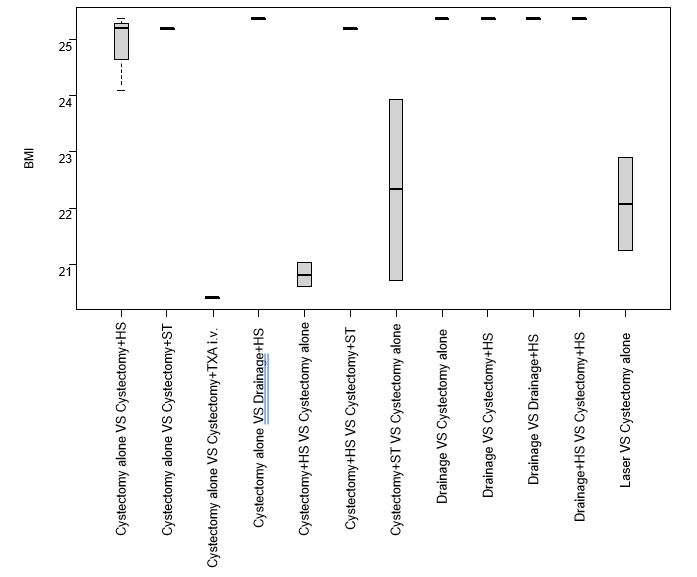


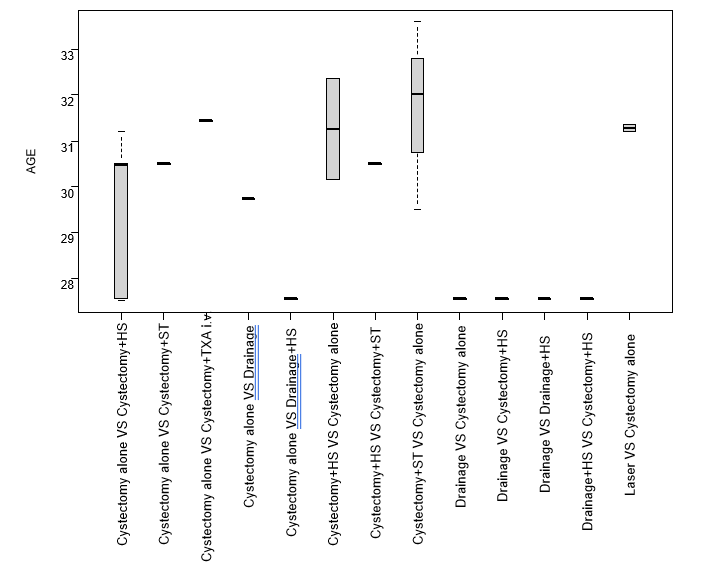


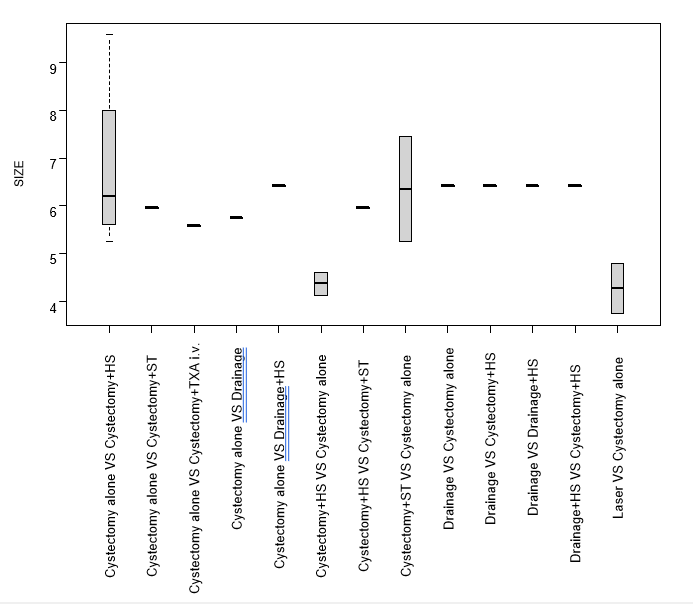


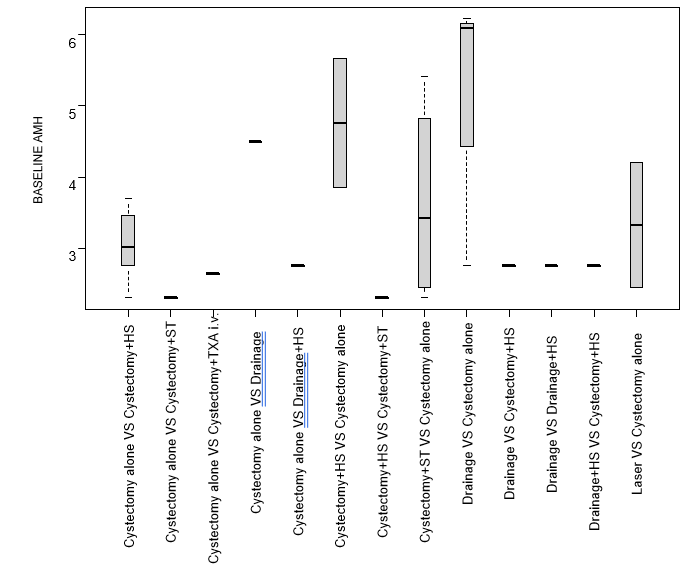


Cystectomy+HS: cystectomy with hemostatic sealants, Cystectomy+TXA i.v.: cystectomy with tranexamic acid, Cystectomy+ST: cystectomy with suture, Drainage+HS: drainage with with hemostatic sealants

# **Supplementary Figure S2.** Risk of bias assessment using revised Cochrane risk-of-bias tool for randomized trials (RoB 2)

| **Study ID** | | | **Weight** | | | | **D1** | | **D2** | | **D3** | **D4** | | | **D5** | | | **Overall** |  |  |  |  | | | | | |  |  |
| --- | --- | --- | --- | --- | --- | --- | --- | --- | --- | --- | --- | --- | --- | --- | --- | --- | --- | --- | --- | --- | --- | --- | --- | --- | --- | --- | --- | --- | --- |
| Shaltout et al., 2019 | | | 1 | | | |  | |  | |  |  | | |  | | |  |  |  |  | Low risk | | | | | |  |  |
| Sweed et al., 2019 | | | 1 | | | |  | |  | |  |  | | |  | | |  |  |  |  | Some concerns | | | | | |  |  |
| Giampaolino et al., 2015 | | | 1 | | | |  |  |  |  |  |  |  |  |  |  |  |  |  |  |  | High risk | | | | | |  |  |
| Candiani et al., 2018 | | | 1 | | | |  | |  | |  |  | | |  | | |  |  |  |  |  | | | | | |  |  |
| Tsolakidis et al., 2010 | | | 1 | | | |  | |  | |  |  | | |  | | |  |  |  |  | |  | |  |  |  |  |  |
| Park et al., 2021 | | | 1 | | | |  |  |  |  |  |  |  |  |  |  |  |  |  |  |  | |  | |  |  |  |  |  |
| Chung et al., 2019 | | | 1 | | | |  | |  | |  |  | | |  | | |  |  |  |  | |  | |  |  |  |  |  |
| Choi et al., 2017 | | | 1 | | | |  | |  | |  |  | | |  | | |  |  |  |  | |  | |  |  |  |  |  |
| Tanprasertkul et al., 2014 | | | 1 | | | |  |  |  |  |  |  |  |  |  |  |  |  |  |  |  | |  | |  |  |  |  |  |
| Asgari et al., 2015 | | | 1 | | | |  | |  | |  |  | | |  | | |  |  |  |  |  | | | | | |  |  |
| Zhang et al., 2016 | | | 1 | | | |  | |  | |  |  | | |  | | |  |  |  |  |  | | | | | |  |  |
| Ferrero et al., 2012 | | | 1 | | | |  |  |  |  |  |  |  |  |  |  |  |  |  |  |  |  | | | | | |  |  |
| Sönmezer et al., 2012 | | | 1 | | | |  | |  | |  |  | | |  | | |  |  |  |  |  | | | | | |  |  |
| Akkaranurakkul et al., 2021 | | | 1 | | | |  | |  | |  |  | | |  | | |  |  |  |  |  | | | | | |  |  |
| Alborzi et al., 2022 | | | 1 | | | |  |  |  |  |  |  |  |  |  |  |  |  |  |  |  |  | | | | | |  |  |
| Javaheri et al., 2021 | | | 1 | | | |  | |  | |  |  | | |  | | |  |  |  |  |  | | | | | |  |  |
| Rouholamin et al., 2019 | | | 1 | | | |  | |  | |  |  | | |  | | |  |  |  |  |  | | | | | |  |  |
| Araujo et al., 2021 | | | 1 | | | |  |  |  |  |  |  |  |  |  |  |  |  |  |  |  |  | | | | | |  |  |
| Ghafarnejad et al., 2014 | | | 1 | | | |  | |  | |  |  | | |  | | |  |  |  |  |  | | | | | |  |  |
| Coric et al., 2011 | | | 1 | | | |  | |  | |  |  | | |  | | |  |  |  |  |  | | | | | |  |  |
| Tehrani et al., 2022 | | | 1 | | | |  |  |  |  |  |  |  |  |  |  |  |  |  |  |  |  | | | | | |  |  |
|  | | |  | | | |  |  |  |  |  |  |  |  |  |  |  |  |  |  |  |  | | | | | |  |  |
|  |  |  | | | |  | |  | |  | | |  |  | |  |  | | | | | | |  | |  |  |  |  |
|  |  |  | |  |  |  |  |  |  |  |  |  |  |  |  |  |  |  |  |  |  |  |  |  |  |  |  |  |  |

D1 Randomisation process

D2 Deviations from the intended interventions

D3 Missing outcome data

D4 Measurement of the outcome

D5 Selection of the reported result

**Supplementary Table S1.** List of excluded studies with reasons

| **1** | Cucinella G, Granese R, Calagna G, et al. Oral contraceptives in the prevention of endometrioma recurrence: does the different progestins used make a difference?. Arch Gynecol Obstet. 2013;288(4):821-827. doi:10.1007/s00404-013-2841-9 | No data on ovarian reserves |
| --- | --- | --- |
| **2** | Muraoka A, Osuka S, Yabuki A, et al. Impact of perioperative use of GnRH agonist or dienogest on ovarian reserve after cystectomy for endometriomas: a randomized controlled trial. Reprod Biol Endocrinol. 2021;19(1):179. Published 2021 Dec 6. doi:10.1186/s12958-021-00866-2 | Only one surgical technique (cystectomy) in both groups |
| **3** | Pellicano M, Bramante S, Guida M, et al. Ovarian endometrioma: postoperative adhesions following bipolar coagulation and suture. Fertil Steril. 2008;89(4):796-799. doi:10.1016/j.fertnstert.2006.11.201 | No data on ovarian reserves |
| **4** | Asgari Z, Moini A, Montazeri A, et al. Comparing the effect of adjunctive N-acetylcysteine plus low dose contraceptive with low dose contraceptive alone on recurrence of ovarian endometrioma and chronic pelvic pain after conservative laparoscopic surgery: a randomised clinical trial study. J Obstet Gynaecol. 2022;42(5):1493-1497. doi:10.1080/01443615.2021.2006165 | No data on ovarian reserves |
| **5** | Chaichian S, Saadat Mostafavi SR, Mehdizadehkashi A, et al. Hyaluronic acid gel application versus ovarian suspension for prevention of ovarian adhesions during laparoscopic surgery on endometrioma: a double-blind randomized clinical trial. BMC Womens Health. 2022;22(1):33. Published 2022 Feb 11. doi:10.1186/s12905-022-01607-2 | No data on ovarian reserves |
| **6** | Fouda UM, Elsetohy KA, Elshaer HS. Barbed Versus Conventional Suture: A Randomized Trial for Suturing the Endometrioma Bed After Laparoscopic Excision of Ovarian Endometrioma. J Minim Invasive Gynecol. 2016;23(6):962-968. doi:10.1016/j.jmig.2016.06.008 | Only one surgical technique (cystectomy with suture) in both groups |
| **7** | Sesti F, Capozzolo T, Pietropolli A, Marziali M, Bollea MR, Piccione E. Recurrence rate of endometrioma after laparoscopic cystectomy: a comparative randomized trial between post-operative hormonal suppression treatment or dietary therapy vs. placebo. Eur J Obstet Gynecol Reprod Biol. 2009;147(1):72-77. doi:10.1016/j.ejogrb.2009.07.003 | No data on ovarian reserves |
| **8** | Seracchioli R, Mabrouk M, Frascà C, et al. Long-term cyclic and continuous oral contraceptive therapy and endometrioma recurrence: a randomized controlled trial. Fertil Steril. 2010;93(1):52-56. doi:10.1016/j.fertnstert.2008.09.052 | No data on ovarian reserves |
| **9** | Demirol A, Guven S, Baykal C, Gurgan T. Effect of endometrioma cystectomy on IVF outcome: a prospective randomized study. Reprod Biomed Online. 2006;12(5):639-643. doi:10.1016/s1472-6483(10)61192-3 | No data on ovarian reserves |
| **10** | Munrós J, Martínez-Zamora MA, Tàssies D, et al. Total Circulating Microparticle Levels After Laparoscopic Surgical Treatment for Endometrioma: A Pilot, Prospective, Randomized Study Comparing Stripping with CO2 Laser Vaporization. J Minim Invasive Gynecol. 2019;26(3):450-455. doi:10.1016/j.jmig.2018.05.014 | No data on ovarian reserves |
| **11** | Seracchioli R, Mabrouk M, Frascà C, Manuzzi L, Savelli L, Venturoli S. Long-term oral contraceptive pills and postoperative pain management after laparoscopic excision of ovarian endometrioma: a randomized controlled trial. Fertil Steril. 2010;94(2):464-471. doi:10.1016/j.fertnstert.2009.03.083 | No data on ovarian reserves |
| **12** | Donnez J, Nisolle M, Gillerot S, Anaf V, Clerckx-Braun F, Casanas-Roux F. Ovarian endometrial cysts: the role of gonadotropin-releasing hormone agonist and/or drainage. Fertil Steril. 1994;62(1):63-66. doi:10.1016/s0015-0282(16)56816-2 | No data on ovarian reserves |
| **13** | Benassi L, Benassi G, Kaihura CT, Marconi L, Ricci L, Vadora E. Chemically assisted dissection of tissues in laparoscopic excision of endometriotic cysts. *J Am Assoc Gynecol Laparosc*. 2003;10(2):205-209. doi:10.1016/s1074-3804(05)60300-3 | No data on ovarian reserves |
| **14** | Muzii L, Achilli C, Bergamini V, et al. Comparison between the stripping technique and the combined excisional/ablative technique for the treatment of bilateral ovarian endometriomas: a multicentre RCT. *Hum Reprod*. 2016;31(2):339-344. doi:10.1093/humrep/dev313 | Two different surgical techniques in the same patient |
| **15** | Hoo WL, Stavroulis A, Pateman K, et al. Does ovarian suspension following laparoscopic surgery for endometriosis reduce postoperative adhesions? An RCT. *Hum Reprod*. 2014;29(4):670-676. doi:10.1093/humrep/deu007 | No data on ovarian reserves |
| **16** | Beretta P, Franchi M, Ghezzi F, Busacca M, Zupi E, Bolis P. Randomized clinical trial of two laparoscopic treatments of endometriomas: cystectomy versus drainage and coagulation. Fertil Steril. 1998;70(6):1176-1180. doi:10.1016/s0015-0282(98)00385-9 | No data on ovarian reserves |
| **17** | Muzii L, Maneschi F, Marana R, et al. Oral estroprogestins after laparoscopic surgery to excise endometriomas: continuous or cyclic administration? Results of a multicenter randomized study. J Minim Invasive Gynecol. 2011;18(2):173-178. doi:10.1016/j.jmig.2010.11.004 | No data on ovarian reserves |
| **18** | Muzii L, Bellati F, Palaia I, et al. Laparoscopic stripping of endometriomas: a randomized trial on different surgical techniques. Part I: clinical results. Hum Reprod. 2005;20(7):1981-1986. doi:10.1093/humrep/dei007 | No data on ovarian reserves |
| **19** | Acién P, Pérez-Albert G, Quereda FJ, Sánchez-Ferrer M, García-Almela A, Velasco I. Treatment of endometriosis with transvaginal ultrasound-guided drainage under GnRH analogues and recombinant interleukin-2 left in the cysts. Gynecol Obstet Invest. 2005;60(4):224-231. doi:10.1159/000089100 | No data on ovarian reserves |
| **20** | Muzii L, Marana R, Caruana P, Catalano GF, Margutti F, Panici PB. Postoperative administration of monophasic combined oral contraceptives after laparoscopic treatment of ovarian endometriomas: a prospective, randomized trial. Am J Obstet Gynecol. 2000;183(3):588-592. doi:10.1067/mob.2000.106817 | No data on ovarian reserves |
| **21** | Velasco I, Campos A, Acién P. Changes in cytokine levels of patients with ovarian endometriosis after treatment with gonadotropin-releasing hormone analogue, ultrasound-guided drainage, and intracystic recombinant interleukin-2. Fertil Steril. 2005;83(4):873-877. doi:10.1016/j.fertnstert.2004.10.035 | No data on ovarian reserves |
| **22** | Graf M, Krüssel JS, Conrad M, Bielfeld P, Rudolf K. Zur Rückbildung funktioneller Zysten: Hochdosierte Ovulationshemmer und Gestagentherapie ohne zusätzlichen Effekt [Regression of functional cysts: high dosage ovulation inhibitor and gestagen therapy has no added effect]. Geburtshilfe Frauenheilkd. 1995;55(7):387-392. doi:10.1055/s-2007-1022807 | No data on ovarian reserves |
| **23** | Rius M, Gracia M, Ros C, et al. Impact of endometrioma surgery on ovarian reserve: a prospective, randomized, pilot study comparing stripping with CO2 laser vaporization in patients with bilateral endometriomas. J Int Med Res. 2020;48(6):300060520927627. doi:10.1177/0300060520927627 | Bilateral endometriomas outcome measured as ovarian volume |
| **24** | Var T, Batioglu S, Tonguc E, Kahyaoglu I. The effect of laparoscopic ovarian cystectomy versus coagulation in bilateral endometriomas on ovarian reserve as determined by antral follicle count and ovarian volume: a prospective randomized study. Fertil Steril. 2011;95(7):2247-2250. doi:10.1016/j.fertnstert.2011.03.078 | Bilateral endometriomas outcome measured as ovarian volume |
| **25** | Asgari Z, Moini A, Montazeri A, et al. Comparing the effect of adjunctive N-acetylcysteine plus low dose contraceptive with low dose contraceptive alone on recurrence of ovarian endometrioma and chronic pelvic pain after conservative laparoscopic surgery: a randomised clinical trial study. J Obstet Gynaecol. 2022;42(5):1493-1497. doi:10.1080/01443615.2021.2006165 | No data on ovarian reserves |
| **26** | Wu Q, Yang Q, Lin Y, Wu L, Lin T. The optimal time for laparoscopic excision of ovarian endometrioma: a prospective randomized controlled trial. Reprod Biol Endocrinol. 2023;21(1):59. Published 2023 Jun 27. doi:10.1186/s12958-023-01109-2 | Laparoscopic cystectomy in both groups |

**Supplementary Table S2.** Characteristics of the included studies

| **Study ID** | | | **Treatment** | **Participants’ characteristics** | | | | **AMH** | | | | | **Endometrioma characteristics** | |  |
| --- | --- | --- | --- | --- | --- | --- | --- | --- | --- | --- | --- | --- | --- | --- | --- |
| **Study number** | **First author** | **Year** | **Treatment name in the network** | **N** | **Drop-outs** | **Mean age**  **(years)** | **Mean BMI**  **(kg/m^2^)** | **kit** | **units** | **Base-line** | **Timing of endpoint result** | **Imputation *** | **Location of OMA** | **Mean size of OMA (cm)** | **Contributes to the network?*** |
| 1 | Shaltout et al., 2019 | 2019 | Drainage | 50 | 3 | 27.55 | 25.37 | AMH Gen II ELISA kits | ng/ml | 2.765 | 6 | none | unilateral unifocal | 6.425 | Yes |
|  |  |  | Cystectomy alone | 50 | 4 |  |  |  |  |  |  |  |  |  |  |
|  |  |  | Drainage+HS | 50 | 5 |  |  |  |  |  |  |  |  |  |  |
|  |  |  | Cystectomy+HS | 50 | 3 |  |  |  |  |  |  |  |  |  |  |
| 2 | Sweed et al., 2019 | 2019 | Drainage | 61 | 0 | 26.3 | 23.10 | ELISA kit, Glory Science Co., Ltd, Del Rio, Texas, USA | ng/ml | 4.225 | NA | none | unilateral and bilateral | 5.35 | Yes |
|  |  |  | Cystectomy alone | 61 | 0 |  |  |  |  |  |  |  |  |  |  |
| 3 | Giampaolino A et al., 2015 | 2015 | Drainage | 15 | 4 | NA | NA | ELISA kit, Diagnostic Systems Laboratories, Webster, TX, USA | ng/ml | 6.225 | 3 | none | unilateral unifocal | >5 | Yes |
|  |  |  | Cystectomy alone | 15 | 4 |  |  |  |  |  |  |  |  |  |  |
| 3 | Giampaolino B et al., 2015 | 2015 | Drainage | 14,5 | 1,5 | NA | NA | ELISA kit, Diagnostic Systems Laboratories, Webster, TX, USA | ng/ml | 6.095 | 3 | none | unilateral unifocal | <5 | Yes |
|  |  |  | Cystectomy alone | 14,5 | 1,5 |  |  |  |  |  |  |  |  |  |  |
| 4 | Candiani et al., 2018 | 2018 | Laser | 30 | 0 | 31.2 | 21.25 | AMH Gen II ELISA, Beckman Coulter Life Sciences, Indiana, USA) | ng/ml | 2.45 | 3 | none | unilateral or bilateral | 4.8 | Yes |
|  |  |  | Cystectomy alone | 30 | 0 |  |  |  |  |  |  |  |  |  |  |
| 5 | Tsolakidis et al., 2010 | 2010 | Laser | 10 | 0 | 31.35 | 22.90 | ELISA kit Diagnostic System Laboratories, webster TX | ng/ml | 4.20 | 6 | Standard deviation | unilateral >3cm | 3.735 | Yes |
|  |  |  | Cystectomy alone | 10 | 0 |  |  |  |  |  |  |  |  |  |  |
| 6 | Park et al., 2021 | 2021 | Cystectomy+HS | 12 | 3 | NA | NA | NR | ng/ml | 3.85 | 3 | none | unilateral unifocal | NA | Yes |
|  |  |  | Cystectomy alone | 10 | 4 |  |  |  |  |  |  |  |  |  |  |
| 7 | Chung et al., 2019 | 2019 | Cystectomy+HS | 47 | 2 | 32.36 | 21.03 | ECLIA Roche Cobas e411 immunoassay analyzer | ng/ml | NA | 3 | Standard deviation | unilateral or bilateral | 4.21 | No |
|  |  |  | Cystectomy alone | 47 | 4 |  |  |  |  |  |  |  |  |  |  |
| 8 | Choi et al., 2017 | 2017 | Cystectomy+HS | 40 | 0 | 30.15 | 20.6 | ELISA kit, Immunotech, Beckman Coulter | ng/ml | 5.665 | 3 | mean | unilateral or bilateral | 4.9 | Yes |
|  |  |  | Cystectomy alone | 40 | 0 |  |  |  |  |  |  |  |  |  |  |
| 9 | Tanprasertkul et al., 2014 | 2014 | Cystectomy+ST | 25 | 0 | 33.60 | 20.7 | ELISA kit Diagnostic System Laboratories, webster TX | ng/ml | 5.420 | 3 and 6 | none | maybe unilateral | NA | Yes |
|  |  |  | Cystectomy alone | 25 | 0 |  |  |  |  |  |  |  |  |  |  |
| 10 | Asgari et al., 2015 | 2015 | Cystectomy+ST | 45 | 0 | 29.51 | 23.94 | ELISA kit, Immunotech, Beckman Coulter | ng/ml | 2.323 | 3 | mean | unilateral unifocal | NA | Yes |
|  |  |  | Cystectomy alone | 47 | 0 |  |  |  |  |  |  |  |  |  |  |
| 11 | Zhang et al., 2016 | 2016 | Cystectomy+ST | 69 | 3 | 32.0 | NA | ELISA kit | ng/ml | 4.250 | 3 and 6 | none | unilateral or bilateral | 5.25 | Yes |
|  |  |  | Cystectomy alone | 69 | 4 |  |  |  |  |  |  |  |  |  |  |
| 12 | Ferrero et al., 2012 | 2012 | Cystectomy+ST | 50 | 8 | 32.0 | NA | ELISA kit Diagnostic System Laboratories, webster TX | ng/ml | 2.588 | 3 and 6 | mean | only bilateral | 7.45 | Yes |
|  |  |  | Cystectomy alone | 50 | 9 |  |  |  |  |  |  |  |  |  |  |
| 13 | Sönmezer et al., 2012 | 2012 | Cystectomy alone | 15 | 0 | 27.5 | 24.1 | ELISA kit, Beckman Coulter Inc., Paris, France | ng/ml | 3.695 | 3 | none | unilateral unifocal | 5.25 | Yes |
|  |  |  | Cystectomy+HS | 15 | 2 |  |  |  |  |  |  |  |  |  |  |
| 14 | Akkaranurakkul et al., 2021 | 2021 | Cystectomy alone | 20 | 0 | 31.45 | 20.4 | ECLIA; Elecsys AMH assay, Roche Diagnostics | ng/ml | 2.650 | 3 | none | unilateral or bilateral | 5.6 | Yes |
|  |  |  | Cystectomy+TXA i.v. | 20 | 0 |  |  |  |  |  |  |  |  |  |  |
| 15 | Alborzi et al., 2022 | 2022 | Cystectomy alone | 51 | 8 | 30.45 | NA | NA | ng/ml | 3.020 | 3 and 6 | none | unilateral unifocal | NA | Yes |
|  |  |  | Cystectomy+HS | 50 | 9 |  |  |  |  |  |  |  |  |  |  |
| 16 | Javaheri et al., 2021 | 2021 | Cystectomy alone | 25 | 0 | 29.73 | NA | ELISA kit | ng/ml | 4.500 | 3 | none | unilateral or bilateral | 5.76 | Yes |
|  |  |  | Drainage | 25 | 0 |  |  |  |  |  |  |  |  |  |  |
| 17 | Rouholamin et al., 2019 | 2019 | Cystectomy alone | 33 | 3 | 31.2 | NA | NA | ng/ml | 3.460 | 6 | none | unilateral unifocal | 9.58 | Yes |
|  |  |  | Cystectomy+HS | 33 | 5 |  |  |  |  |  |  |  |  |  |  |
| 18 | Araujo et al., 2021 | 2021 | Cystectomy alone | 28 | 1 | 30.5 | 25.2 | ELISA kit Diagnostic System Laboratories, webster TX | ng/ml | 2.305 | 6 | none | unilateral unifocal | 5.96 | Yes |
|  |  |  | Cystectomy+HS | 28 | 4 |  |  |  |  |  |  |  |  |  |  |
|  |  |  | Cystectomy+ST | 28 | 2 |  |  |  |  |  |  |  |  |  |  |
| 19 | Ghafarnejad et al., 2014 | 2014 | Cystectomy alone | 10 | 4 | 27.25 | 22.25 | NA | NA | NA | NA | none | unilateral unifocal | 5.145 | No |
|  |  |  | Cystectomy+HS | 10 | 4 |  |  |  |  |  |  |  |  |  |  |
| 20 | Coric et al., 2011 | 2011 | Cystectomy alone | 25 | 3 | 30.3 | NA | NA | NA | NA | NA | none | unilateral unifocal | 4.26 | No |
|  |  |  | Cystectomy+ST | 25 | 2 |  |  |  |  |  |  |  |  |  |  |
| 21 | Tehrani et al., 2022 | 2022 | Cystectomy alone | 37 | 2 | 31.46 | 23.12 | NA | NA | NA | NA | none | unilateral or bilateral | NA | No |
|  |  |  | Sclerotherapy | 36 | 1 |  |  |  |  |  |  |  |  |  |  |

Cystectomy+HS: cystectomy with hemostatic sealants, Cystectomy+TXA i.v.: cystectomy with tranexamic acid, Cystectomy+ST: cystectomy with suture, Drainage+HS: drainage with with hemostatic sealants

# **Supplementary Table S3.** Description of the interventions framed following the network nodes classification

| Study | Surgery description | Name in the network |
| --- | --- | --- |
| Shaltout et al., 2019 | laparoscopic fenestration and electrocautery of the endometrioma cyst wall  laparoscopic excision of the endometrioma cyst wall  laparoscopic fenestration of the endometrioma cyst wall followed by insertion of 4–8 pieces of Surgicel inside the cyst cavity  laparoscopic excision of the endometrioma cyst wall followed by insertion of 4–8 pieces of Surgicel inside the remaining ovarian tissues. | Drainage  Cystectomy alone  Drainage+HS  Cystectomy+HS |
| Sweed et al., 2019 | laparoscopic cyst deroofing  laparoscopic ovarian cystectomy | Drainage  Cystectomy alone |
| Giampaolino et al., 2015 | laparoscopic ovarian cyst wall removal, when necessary minimal bipolar coagulation was used to control significant bleeding. After cystectomy, the ovarian cortex was closed using an intracorporeal stitch  laparoscopic cyst fenestration, biopsy sample, coagulation of the inner cyst wall using bipolar forceps. Ovarian edges were closed by suture. | Cystectomy alone  Drainage |
| Candiani et al., 2018 | standardized laparoscopic stripping technique  drainage of the cyst content, irrigation and inspection of its inner wall, biopsy, the cyst was everted and completely vaporized with CO2 fiber laser (UltraPulse Duo system, Lumenis Ltd, Yokneam, Israel) in a radial way starting from the center to the periphery, at a power density of 13 W/cm 2. No suture was placed after vaporization. | Cystectomy alone  Laser |
| Tsolakidis et al., 2010 | Laparoscopic ovarian cystectomy with bipolar energy. No sutures placed.  Three-step technique: During the first laparoscopy only drainage and biopsy, then GnRH agonists were administered for 3 months and a second laparoscopy was carried out to vaporize the internal wall by using a CO 2 laser (Sharplan 1041S; Shar- plan, Tel-Aviv, Israel) at a power density of 14,000 W/cm2 . No sutures placed. | Cystectomy alone  Laser |
| Park et al., 2021 | Laparoscopic ovarian cystectomy and use of SurgiGuard. If hemostasis failed within 10 min bipolar coagulation, and suturing if needed, were conducted.  Laparoscopic ovarian cystectomy with bipolar coagulation. If hemostasis failed within 10 min, suturing was performed. | Cystectomy+HS  Cystectomy alone |
| Chung et al., 2019 | Laparoscopic ovarian cystectomy with diathermy  Laparoscopic ovarian cystectomy with use of FloSeal | Cystectomy alone  Cystectomy+HS |
| Choi et al., 2017 | Laparoscopic ovarian cystectomy with electrocoagulation  Laparoscopic ovarian cystectomy with use of FloSeal or TachoSil | Cystectomy alone  Cystectomy+HS |
| Tanprasertkul et al., 2014 | Laparoscopic ovarian cystectomy with electrocoagulation  Laparoscopic ovarian cystectomy with suture | Cystectomy alone  Cystectomy+ST |
| Asgari et al., 2015 | Laparoscopic ovarian cystectomy with bipolar coagulation  Laparoscopic ovarian cystectomy with suture | Cystectomy alone  Cystectomy+ST |
| Zhang et al., 2016 | Laparoscopic ovarian cystectomy with bipolar electrocoagulation  Laparoscopic ovarian cystectomy with suture | Cystectomy alone  Cystectomy+ST |
| Ferrero et al., 2012 | Laparoscopic ovarian cystectomy with bipolar electrocoagulation  Laparoscopic ovarian cystectomy with suture | Cystectomy alone  Cystectomy+ST |
| Sönmezer et al., 2012 | Laparoscopic ovarian cystectomy with bipolar electrocoagulation  Laparoscopic ovarian cystectomy with hemostatic matrix | Cystectomy alone  Cystectomy+HS |
| Akkaranurakkul et al., 2021 | Laparoscopic ovarian cystectomy with bipolar electrocoagulation  Laparoscopic ovarian cystectomy with bipolar electrocoagulation and TXA 1 g intravenously within 10 min before making skin incision. | Cystectomy alone  Cystectomy+TXA |
| Alborzi et al., 2022 | Laparoscopic ovarian cystectomy with bipolar electrocoagulation  Laparoscopic ovarian cystectomy with vasopressin injection | Cystectomy alone  Cystectomy+HS |
| Javaheri et al., 2021 | Laparoscopic ovarian cystectomy with bipolar electrocoagulation  Laparoscopic ovarian partial cystectomy; hemostasis with electrocoagulation | Cystectomy alone  Drainage |
| Rouholamin et al., 2019 | Laparoscopic ovarian cystectomy  Laparoscopic ovarian cystectomy with vasopressin injection | Cystectomy alone  Cystectomy+HS |
| Araujo et al., 2021 | Laparoscopic ovarian cystectomy with bipolar coagulation  Laparoscopic ovarian cystectomy with hemostatic sealants  Laparoscopic ovarian cystectomy with suture | Cystectomy alone  Cystectomy+HS  Cystectomy+ST |
| Ghafarneja et al., 2014 | Laparoscopic ovarian cystectomy with bipolar electrocoagulation  Laparoscopic ovarian cystectomy with vasopressin injection | Cystectomy alone  Cystectomy+HS |
| Coric et al., 2011 | Laparoscopic ovarian cystectomy with bipolar electrocoagulation  Laparoscopic ovarian cystectomy with suture | Cystectomy alone  Cystectomy+ST |
| Tehrani et al., 2022 | Laparoscopic ovarian cystectomy with bipolar electrocoagulation  Transvaginal sclerotherapy with 100ml of 95% ethanol which remained in the cyst for 20 minutes | Cystectomy alone  Sclerotherapy |

Cystectomy+HS: cystectomy with hemostatic sealants, Cystectomy+TXA i.v.: cystectomy with tranexamic acid, Cystectomy+ST: cystectomy with suture, Drainage+HS: drainage with with hemostatic sealant

**Supplementary Table S4.** Certainty of evidence according to CiNEMA assessment

Cystectomy+HS: cystectomy with hemostatic sealants, Cystectomy+TXA i.v.: cystectomy with tranexamic acid, Cystectomy+ST: cystectomy with suture, Drainage+HS: drainage with with hemostatic sealants

**Supplementary Table S5.** Subgroup analyses

| Subgroup | Analysis | Network map | Forest plot | Heterogeneity | inconsistency |
| --- | --- | --- | --- | --- | --- |
| Size of endometrioma | <5cm | N= 3 n=208  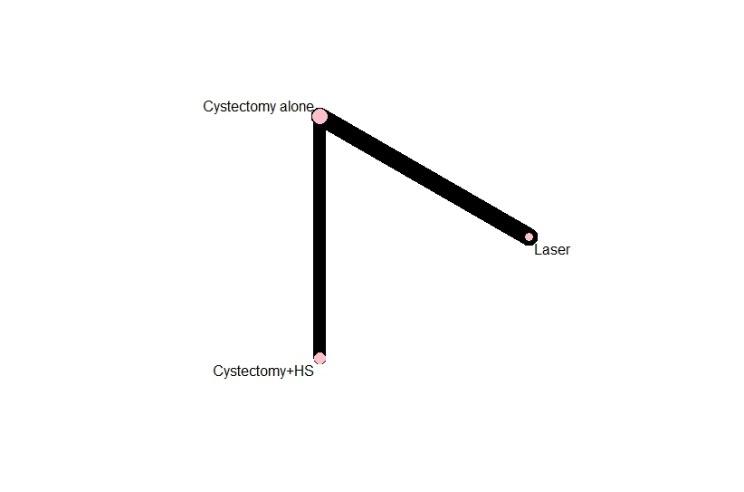 | 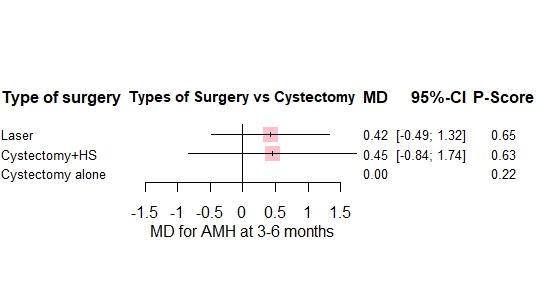 | τ^2^= 0.266  I^2^=1% | Global:  p= NA  Local (SIDE):  0/0 |
|  | >5cm | N=11 n=707  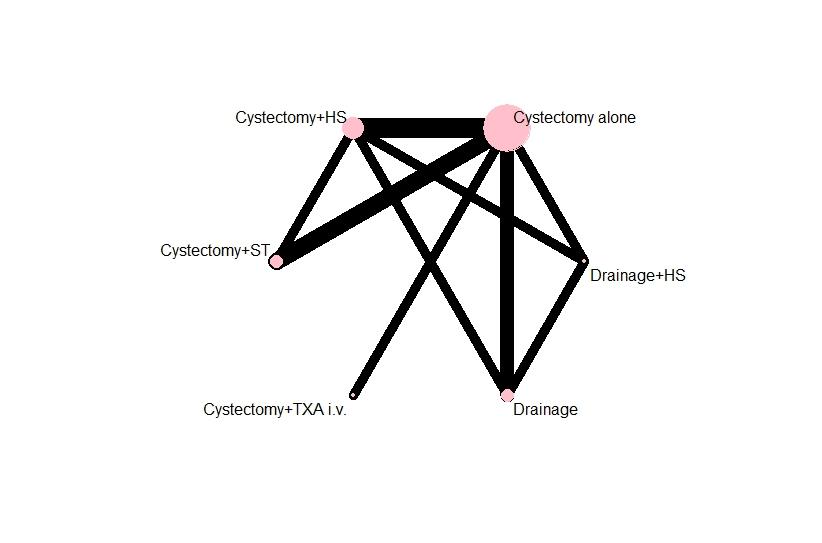 | 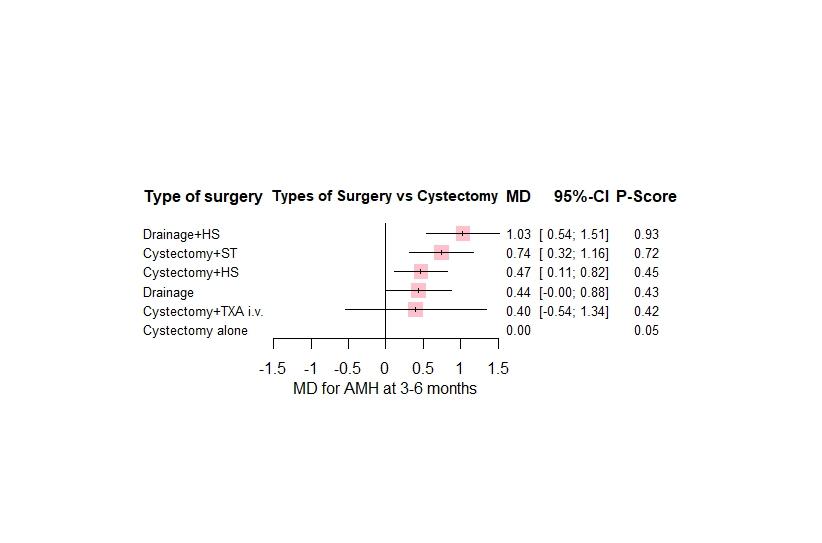 | τ^2^=0.0567  I^2^=0% | Global:  p=0.222  Local (SIDE):  0/8 |
| Timing of AMH measurement | 3 months | N=13 n=857  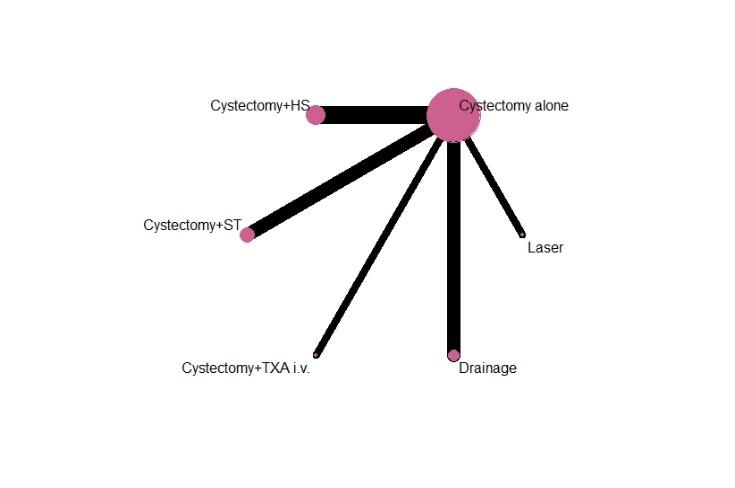 | 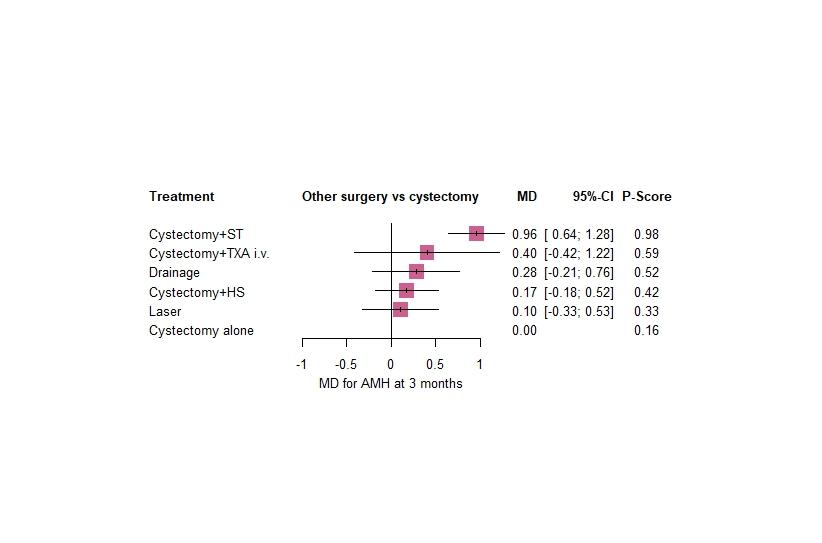 | τ^2^=NA  I^2^=NA | Global:  p=NA  Local (SIDE):  0/0 |
|  | 6 months | N=8 n=648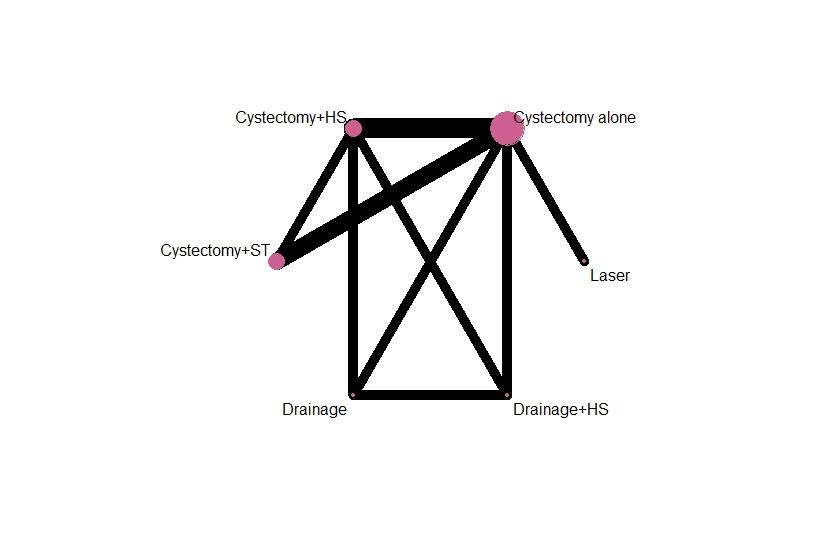 | 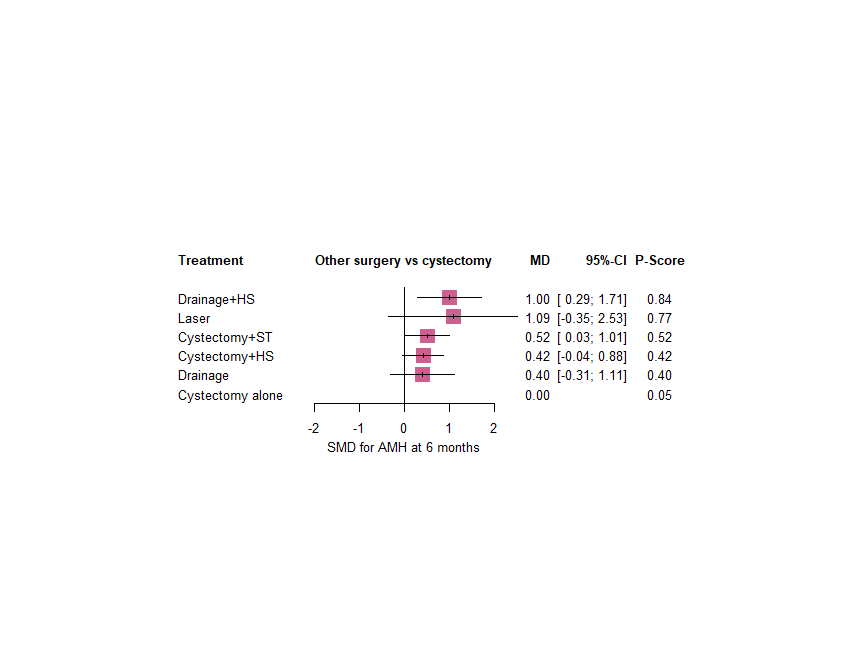 | τ^2^=0.1388  I^2^=1% | Global:  p=NA  Local (SIDE):  0/7 |
| Localization of endometrioma | Only unilateral | N=10 n=1110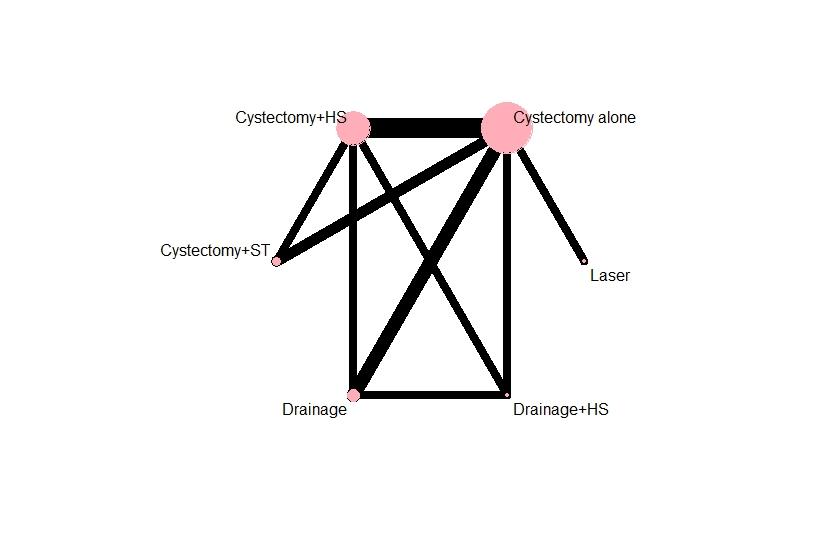 | 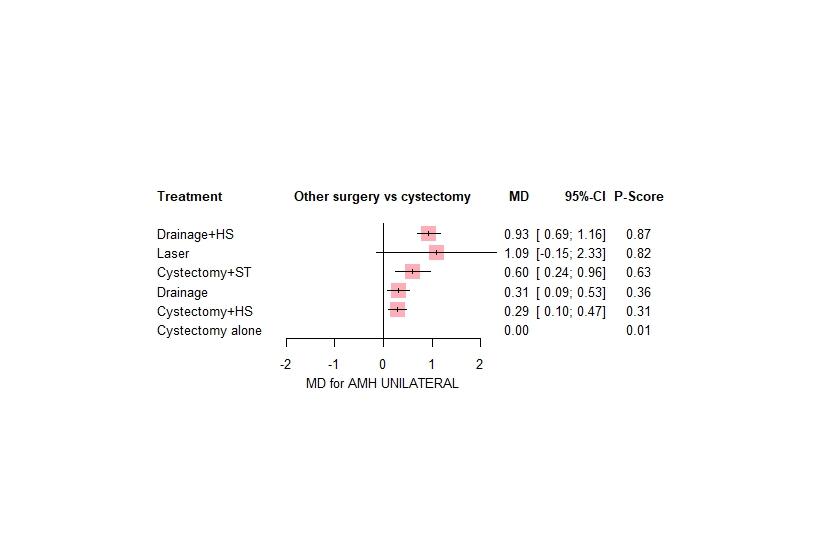 | τ^2^=0  I^2^=0 | Global:  p=0.354  Local (SIDE):  2/8 |
|  | Bilateral and unilateral | N=6 n=401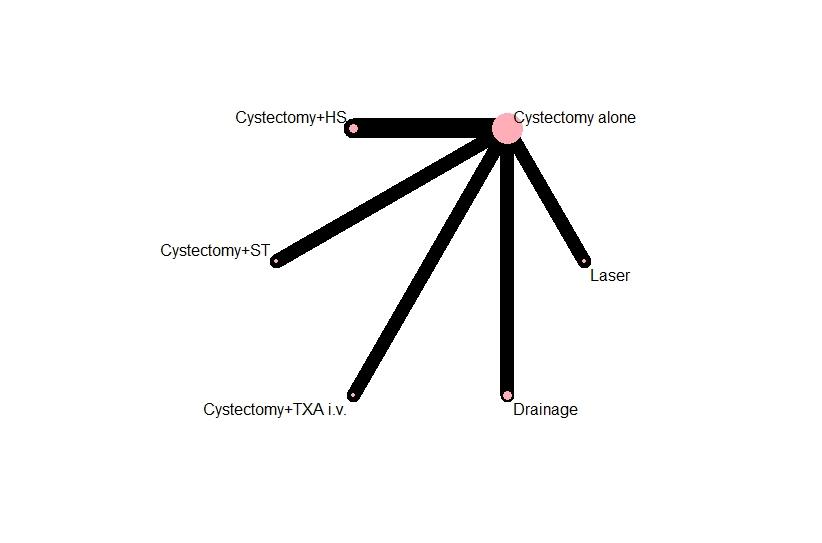 | 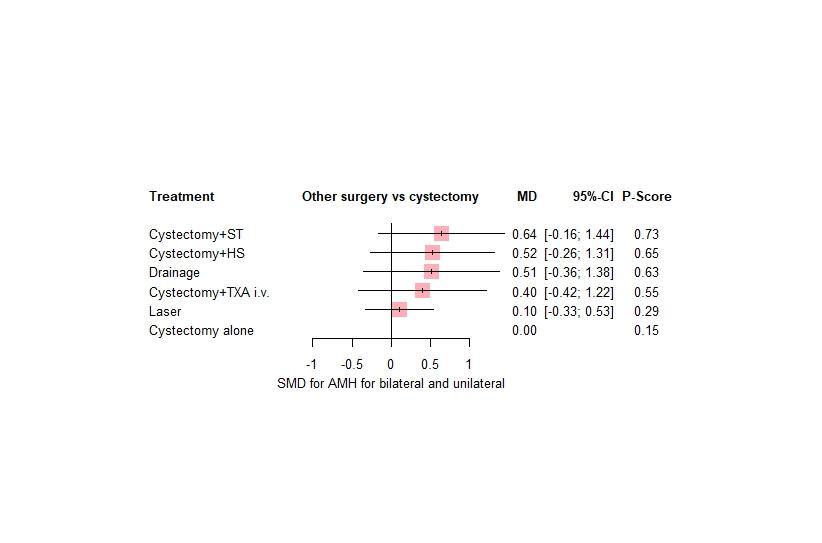 | τ^2^=0  I^2^=0 | Global:  p=NA  Local (SIDE):  0/0 |

Cystectomy+HS: cystectomy with hemostatic sealants, Cystectomy+TXA i.v.: cystectomy with tranexamic acid, Cystectomy+ST: cystectomy with suture, Drainage+HS: drainage with with hemostatic sealants

**Supplementary File S1.** Search strategy

(endometrio*) AND ((cystectomy) OR (laser) OR (plasma) OR (sclerotherapy) OR (drainage) OR (cystotomy) OR (surgical therapy) OR (expectative))

# **Supplementary File S2.** Additional results of the network meta-analysis of the primary outcome: AMH at endpoint (3-6 months)

## **Characteristics of the network**

- Number of studies: k= 18
- Number of pairwise comparisons: m=25
- Number of treatments: n= 7
- Number of designs d=7

## **Results of statistical test for inconsistency of the network and common estimate for heterogeneity**

| **Inconsistent comparisons of detachable comparisons (%) (SIDE-test)** | **P-value of Design-by-treatment test** | **Common-Tau (standard deviation of differences in effect size between studies of the same comparison)** |
| --- | --- | --- |
| 1 of 8 (12.5%) | 0.581 | 0.16 |

## **Forest plot with results of the pairwise meta-analyses**

##
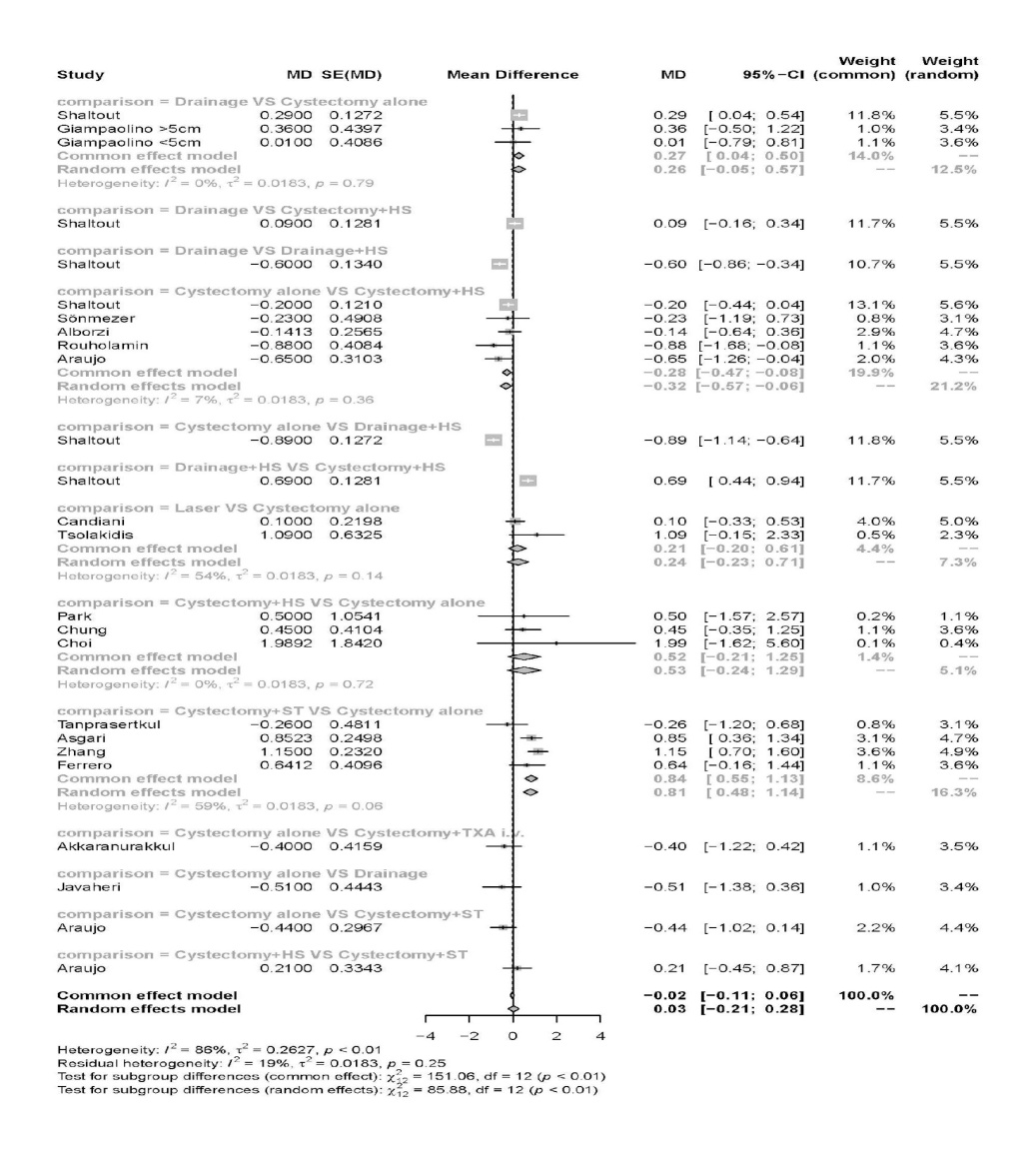


Cystectomy+HS: cystectomy with hemostatic sealants, Cystectomy+TXA i.v.: cystectomy with tranexamic acid, Cystectomy+ST: cystectomy with suture, Drainage+HS: drainage with with hemostatic sealants

# **Supplementary File S3.** Sensitivity analyses

## **High quality studies only**

Number of studies: k = 15

Number of pairwise comparisons: m = 22

Number of treatments: n = 7

Number of designs: d = 7

**Network-plot**

**
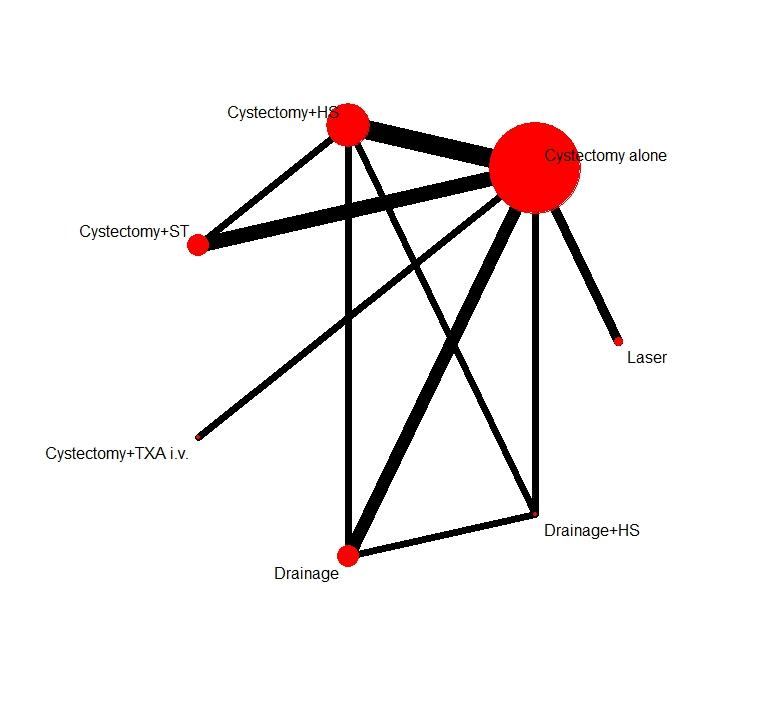
**

Lines link treatments with direct comparisons in trials; thickness of lines corresponds to the number of trials evaluating the comparison; size of the nodes corresponds to the number of participants assigned to the treatment. Cystectomy+HS: cystectomy with hemostatic sealants, Cystectomy+TXA i.v.: cystectomy with tranexamic acid, Cystectomy+ST: cystectomy with suture, Drainage+HS: drainage with with hemostatic sealants

**Forest-plot of results of the network-meta-analysis (reference cystectomy alone)**

**
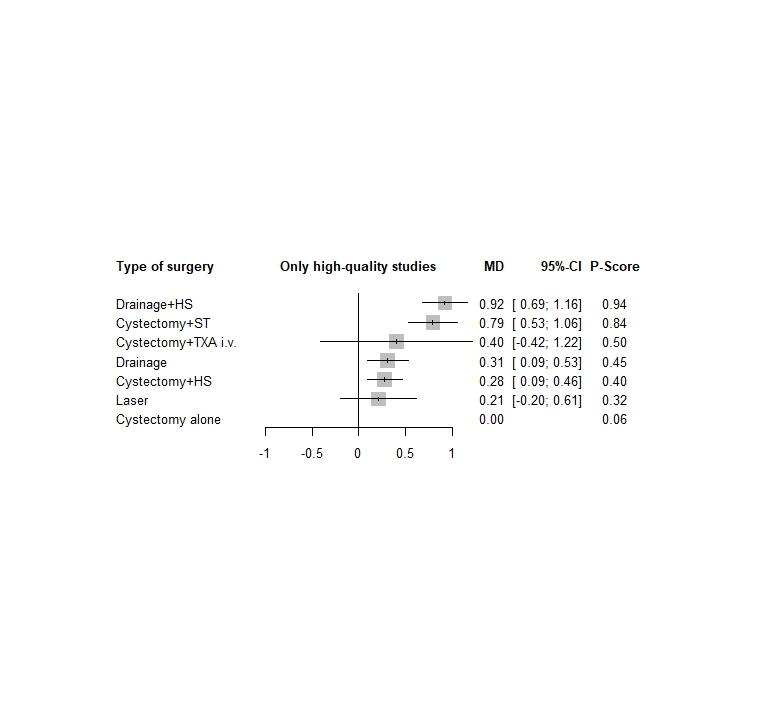
**

MD= mean difference; CI=confidence intervals

Cystectomy+HS: cystectomy with hemostatic sealants, Cystectomy+TXA i.v.: cystectomy with tranexamic acid, Cystectomy+ST: cystectomy with suture, Drainage+HS: drainage with with hemostatic sealants

**League table**

| Drainage+HS | . | . | 0.60 [ 0.34; 0.86] | 0.69 [ 0.44; 0.94] | . | 0.89 [ 0.64; 1.14] |
| --- | --- | --- | --- | --- | --- | --- |
| 0.13 [-0.22; 0.48] | Cystectomy+ST | . | . | -0.21 [-0.87; 0.45] | . | 0.84 [ 0.57; 1.12] |
| 0.52 [-0.33; 1.37] | 0.39 [-0.46; 1.25] | Cystectomy+TXA i.v. | . | . | . | 0.40 [-0.42; 1.22] |
| **0.61 [ 0.36; 0.87]** | **0.49 [ 0.14; 0.83]** | 0.09 [-0.75; 0.94] | Drainage | 0.09 [-0.16; 0.34] | . | 0.27 [ 0.04; 0.50] |
| **0.65 [ 0.41; 0.89]** | **0.52 [ 0.20; 0.83]** | 0.12 [-0.71; 0.96] | 0.03 [-0.20; 0.26] | Cystectomy+HS | . | 0.26 [ 0.07; 0.45] |
| **0.71 [ 0.24; 1.19]** | **0.59 [ 0.10; 1.07]** | 0.19 [-0.72; 1.10] | 0.10 [-0.36; 0.56] | 0.07 [-0.38; 0.52] | Laser | 0.21 [-0.20; 0.61] |
| **0.92 [ 0.69; 1.16]** | **0.79 [ 0.53; 1.06]** | 0.40 [-0.42; 1.22] | **0.31 [ 0.09; 0.53]** | **0.28 [ 0.09; 0.46]** | 0.21 [-0.20; 0.61] | Cystectomy alone |

Cystectomy+HS: cystectomy with hemostatic sealants, Cystectomy+TXA i.v.: cystectomy with tranexamic acid, Cystectomy+ST: cystectomy with suture, Drainage+HS: drainage with with hemostatic sealants

**Results of statistical test for inconsistency of the network and common estimate for heterogeneity**

| **Inconsistent comparisons of detachable comparisons (%) (SIDE-test)** | **P-value of Design-by-treatment test** | **Common-Tau (standard deviation of differences in effect size between studies of the same comparison)** |
| --- | --- | --- |
| 2 of 8 (25%) | 0.167 |  |

## **Studies with no imputations only**

Number of studies: k = 13

Number of pairwise comparisons: m = 20

Number of treatments: n = 7

Number of designs: d = 7

**Network-plot**

**
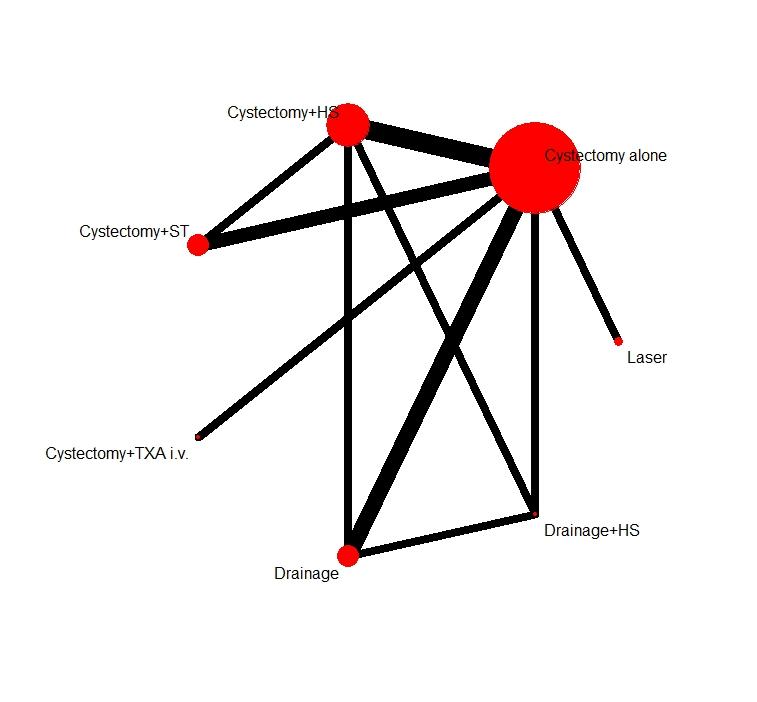
**

Lines link treatments with direct comparisons in trials; thickness of lines corresponds to the number of trials evaluating the comparison; size of the nodes corresponds to the number of participants assigned to the treatment

Cystectomy+HS: cystectomy with hemostatic sealants, Cystectomy+TXA i.v.: cystectomy with tranexamic acid, Cystectomy+ST: cystectomy with suture, Drainage+HS: drainage with with hemostatic sealants

**Forest-plot of results of the network-meta-analysis (reference cystectomy alone)**

**
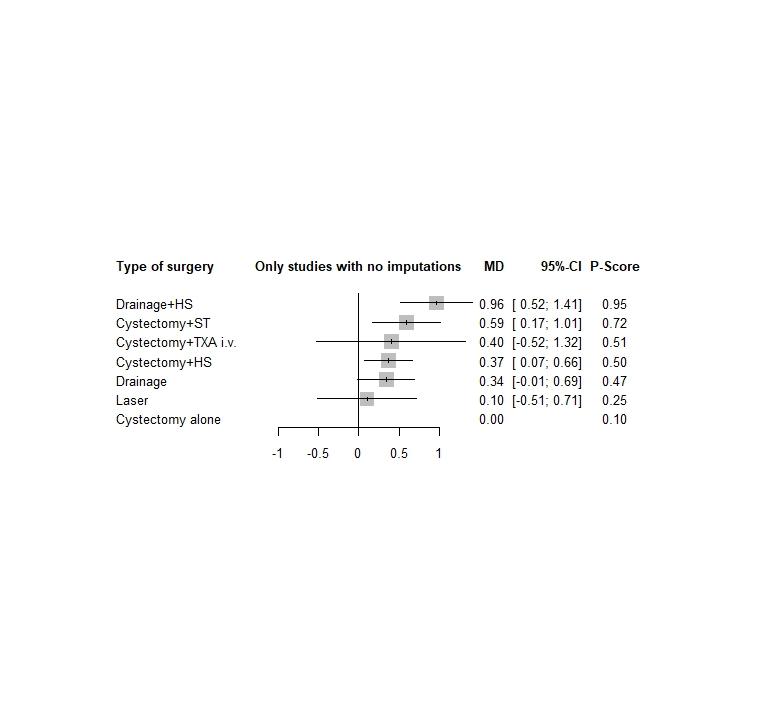
**

MD= mean difference; CI=confidence intervals

Cystectomy+HS: cystectomy with hemostatic sealants, Cystectomy+TXA i.v.: cystectomy with tranexamic acid, Cystectomy+ST: cystectomy with suture, Drainage+HS: drainage with with hemostatic sealants

**League table**

| Drainage+HS | . | . | 0.60 [ 0.10; 1.10] | 0.69 [ 0.19; 1.19] | . | 0.89 [ 0.39; 1.39] |
| --- | --- | --- | --- | --- | --- | --- |
| 0.37 [-0.23; 0.97] | Cystectomy+ST | . | . | -0.21 [-0.99; 0.57] | . | 0.66 [ 0.22; 1.09] |
| 0.56 [-0.46; 1.58] | 0.19 [-0.82; 1.20] | Cystectomy+TXA i.v. | . | . | . | 0.40 [-0.52; 1.32] |
| **0.62 [ 0.15; 1.10]** | 0.25 [-0.28; 0.79] | 0.06 [-0.92; 1.05] | Drainage | 0.09 [-0.41; 0.59] | . | 0.29 [-0.08; 0.65] |
| **0.59 [ 0.14; 1.05]** | 0.22 [-0.26; 0.71] | 0.03 [-0.94; 1.00] | -0.03 [-0.43; 0.37] | Cystectomy+HS | . | 0.35 [ 0.04; 0.66] |
| **0.86 [ 0.11; 1.62]** | 0.49 [-0.25; 1.23] | 0.30 [-0.80; 1.40] | 0.24 [-0.47; 0.94] | 0.27 [-0.41; 0.94] | Laser | 0.10 [-0.51; 0.71] |
| **0.96 [ 0.52; 1.41]** | **0.59 [ 0.17; 1.01]** | 0.40 [-0.52; 1.32] | 0.34 [-0.01; 0.69] | **0.37 [ 0.07; 0.66]** | 0.10 [-0.51; 0.71] | Cystectomy alone |

Cystectomy+HS: cystectomy with hemostatic sealants, Cystectomy+TXA i.v.: cystectomy with tranexamic acid, Cystectomy+ST: cystectomy with suture, Drainage+HS: drainage with with hemostatic sealants

**Results of statistical test for inconsistency of the network and common estimate for heterogeneity**

| **Inconsistent comparisons of detachable comparisons (%) (SIDE-test)** | **P-value of Design-by-treatment test** | **Common-Tau (standard deviation of differences in effect size between studies of the same comparison)** |
| --- | --- | --- |
| 0 of 8 (0%) | 0.902 | 0.22 |

## **Studies after exclusion of the studies with high baseline AMH levels**

Number of studies: k = 15

Number of pairwise comparisons: m = 22

Number of treatments: n = 7

Number of designs: d = 8

**Network-plot**


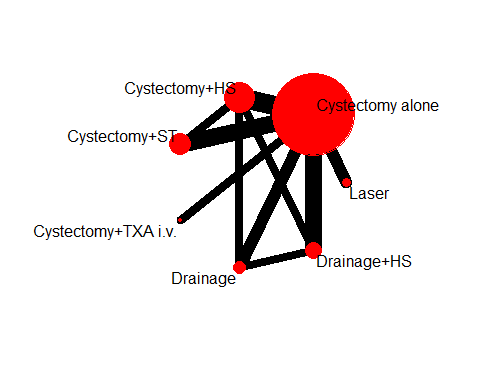


Lines link treatments with direct comparisons in trials; thickness of lines corresponds to the number of trials evaluating the comparison; size of the nodes corresponds to the number of participants assigned to the treatment

Cystectomy+HS: cystectomy with hemostatic sealants, Cystectomy+TXA i.v.: cystectomy with tranexamic acid, Cystectomy+ST: cystectomy with suture, Drainage+HS: drainage with with hemostatic sealants

**Forest-plot of results of the network-meta-analysis (reference cystectomy alone)**

**
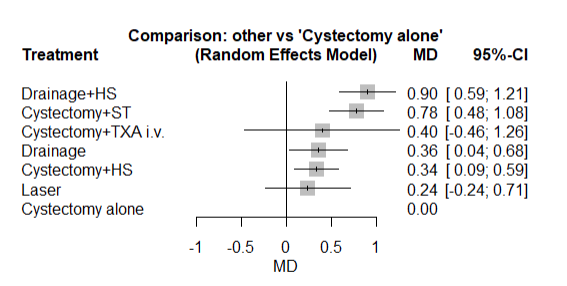
**

MD= mean difference; CI=confidence intervals

Cystectomy+HS: cystectomy with hemostatic sealants, Cystectomy+TXA i.v.: cystectomy with tranexamic acid, Cystectomy+ST: cystectomy with suture, Drainage+HS: drainage with with hemostatic sealants

**Results of statistical test for inconsistency of the network and common estimate for heterogeneity**

| **Inconsistent comparisons of detachable comparisons (%) (SIDE-test)** | **P-value of Design-by-treatment test** | **Common-Tau (standard deviation of differences in effect size between studies of the same comparison)** |
| --- | --- | --- |
| 2 of 8 (25%) | 0.184 | 0.138 |

# **Supplementary File S4.** Network plot of secondary outcome “AFC at endpoint (3-6 months)

Number of studies: k = 8

Number of pairwise comparisons: m = 13

Number of treatments: n = 6

Number of designs: d = 5

**Network-plot**


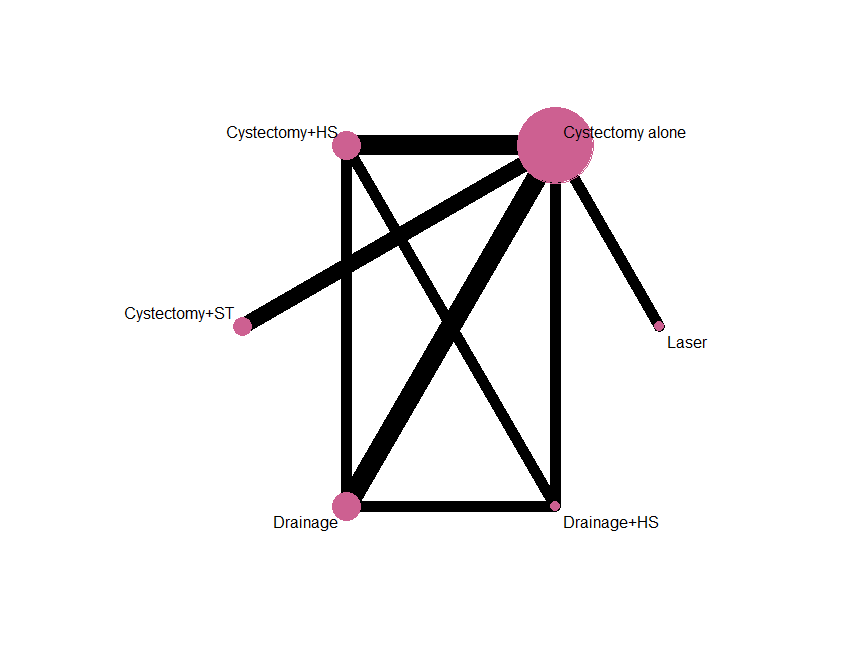


Lines link treatments with direct comparisons in trials; thickness of lines corresponds to the number of trials evaluating the comparison; size of the nodes corresponds to the number of participants assigned to the treatment

Cystectomy+HS: cystectomy with hemostatic sealants, Cystectomy+TXA i.v.: cystectomy with tranexamic acid, Cystectomy+ST: cystectomy with suture, Drainage+HS: drainage with with hemostatic sealants

**Forest-plot of results of the network-meta-analysis (reference cystectomy alone)**


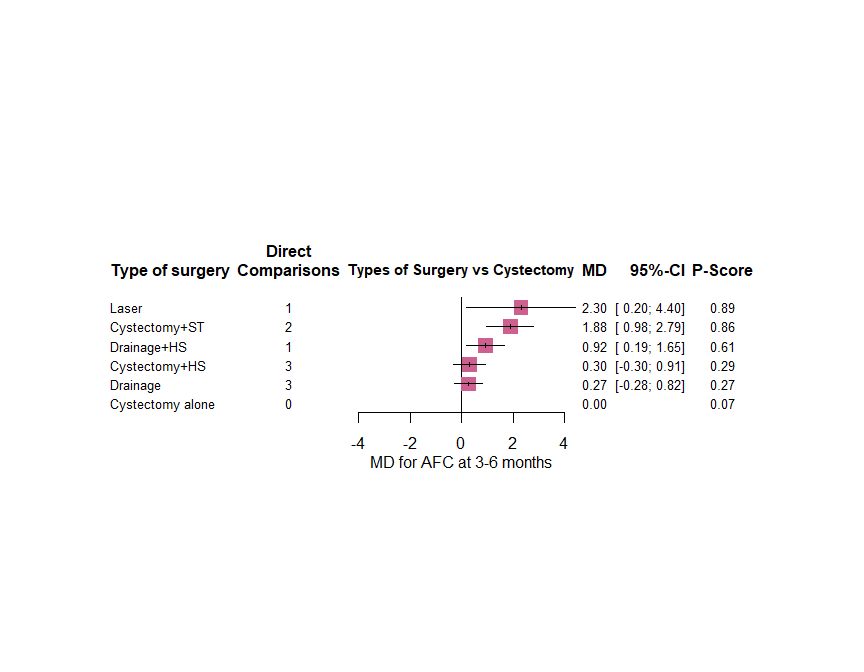


MD= mean difference; CI=confidence intervals

Cystectomy+HS: cystectomy with hemostatic sealants, Cystectomy+TXA i.v.: cystectomy with tranexamic acid, Cystectomy+ST: cystectomy with suture, Drainage+HS: drainage with with hemostatic sealants

**League table**

| **Laser** | **.** | **.** | **.** | **.** | **2.30 [ 0.20; 4.40]** |
| --- | --- | --- | --- | --- | --- |
| **0.42 [-1.87; 2.70]** | **Cystectomy+ST** | **.** | **.** | **.** | **1.88 [ 0.98; 2.79]** |
| **1.38 [-0.84; 3.60]** | **0.97 [-0.20; 2.13]** | **Drainage+HS** | **0.69 [-0.11; 1.49]** | **0.60 [-0.20; 1.40]** | **0.89 [ 0.09; 1.69]** |
| **2.00 [ -0.19; 4.18]** | **1.58 [ 0.49; 2.67]** | **0.61 [-0.15; 1.38]** | **Cystectomy+HS** | **-0.09 [-0.89; 0.71]** | **0.33 [-0.30; 0.97]** |
| **2.03 [-0.14; 4.20]** | **1.61 [ 0.55; 2.68]** | **0.65 [-0.10; 1.40]** | **0.03 [-0.65; 0.71]** | **Drainage** | **0.23 [-0.33; 0.80]** |
| **2.30 [ 0.20; 4.40]** | **1.88 [ 0.98; 2.79]** | **0.92 [ 0.19; 1.65]** | **0.30 [-0.30; 0.91]** | **0.27 [-0.28; 0.82]** | **Cystectomy alone** |

Network plot of primary outcome “AFC at 3-6 months after surgery”. The lines link interventions that were directly compared in trials. The thickness of the lines corresponds to the number of studies evaluating the comparison. The size of the nodes corresponds to the number of participants assigned to the intervention. b) Forest plot of different techniques for endometrioma vs. cystectomy alone for the secondary outcome “AFC at 3-6 months after surgery”. Outcomes are reported as mean differences (MDs) and 95% confidence interval (CIs). b) League table for the secondary outcome “AFC at 3-6 months after surgery”.

Cystectomy+HS: cystectomy with hemostatic sealants, Cystectomy+TXA i.v.: cystectomy with tranexamic acid, Cystectomy+ST: cystectomy with suture, Drainage+HS: drainage with with hemostatic sealants

# **Supplementary File S5.** Differences between protocol and review

The initial PROSPERO protocol (registration number: CRD42021238909) was designed to be as broad as possible and include different study designs (RCTs and observational studies) as well as several surgical and reproductive outcomes. To include all the data in one meta-analysis would be impossible so we decided to report the data in several studies. For the current meta-analysis, which is the first according to the above prospero project, we included only the highest research design (RCT) and as a primary outcome the AMH since this outcome is the most frequently reported reproductive outcome and thus having the possibility to include the maximum of studies and obtain results of high clinical significant.
